# Gene networks and transcription factor motifs defining the differentiation of stem cells into hepatocyte-like cells

**DOI:** 10.1016/j.jhep.2015.05.013

**Published:** 2015-10

**Authors:** Patricio Godoy, Wolfgang Schmidt-Heck, Karthick Natarajan, Baltasar Lucendo-Villarin, Dagmara Szkolnicka, Annika Asplund, Petter Björquist, Agata Widera, Regina Stöber, Gisela Campos, Seddik Hammad, Agapios Sachinidis, Umesh Chaudhari, Georg Damm, Thomas S. Weiss, Andreas Nüssler, Jane Synnergren, Karolina Edlund, Barbara Küppers-Munther, David C. Hay, Jan G. Hengstler

**Affiliations:** 1 IfADo-Leibniz Research Centre for Working Environment and Human Factors at the Technical University Dortmund, Dortmund, Germany; 2 Leibniz Institute for Natural Product Research and Infection Biology eV-Hans-Knöll Institute, Jena, Germany; 3 University of Cologne, Institute of Neurophysiology and Center for Molecular Medicine Cologne (CMMC), Robert-Koch-Str. 39, 50931 Cologne, Germany; 4 MRC Centre for Regenerative Medicine, University of Edinburgh, Edinburgh EH16 4UU, United Kingdom; 5 Takara Bio Europe AB (former Cellartis AB), Arvid Wallgrens Backe 20, 41346 Gothenburg, Sweden; 6 Systems Biology Research Center, School of Bioscience, University of Skövde, Sweden; 7 NovaHep AB, Arvid Wallgrens Backe 20, 41346 Gothenburg, Sweden; 8 Charité University Medicine Berlin, Department of General-, Visceral- and Transplantation Surgery, D13353 Berlin, Germany; 9 Center for Liver Cell Research, Department of Pediatrics and Juvenile Medicine, University of Regensburg Hospital, Regensburg, Germany; 10 Eberhard Karls University Tübingen, BG Trauma Center, Siegfried Weller Institut, D72076 Tübingen, Germany; 11 Department of Physiology, Faculty of Biological Sciences, University of Concepción, Chile

**Keywords:** FH, Freshly isolated hepatocytes, CS, collagen sandwich, CM, collagen monolayer, SC, stem cells, HLC, hepatocyte-like cells, TF, transcription factors, Stem cells, Hepatocytes, Differentiation, Gene array, Bioinformatics, Transcriptomics, Gene networks

## Abstract

**Background & Aims:**

The differentiation of stem cells to hepatocyte-like cells (HLC) offers the perspective of unlimited supply of human hepatocytes. However, the degree of differentiation of HLC remains controversial. To obtain an unbiased characterization, we performed a transcriptomic study with HLC derived from human embryonic and induced stem cells (ESC, hiPSC) from three different laboratories.

**Methods:**

Genome-wide gene expression profiles of ESC and HLC were compared to freshly isolated and up to 14 days cultivated primary human hepatocytes. Gene networks representing successful and failed hepatocyte differentiation, and the transcription factors involved in their regulation were identified.

**Results:**

Gene regulatory network analysis demonstrated that HLC represent a mixed cell type with features of liver, intestine, fibroblast and stem cells. The “unwanted” intestinal features were associated with KLF5 and CDX2 transcriptional networks. Cluster analysis identified highly correlated groups of genes associated with mature liver functions (n = 1057) and downregulated proliferation associated genes (n = 1562) that approach levels of primary hepatocytes. However, three further clusters containing 447, 101, and 505 genes failed to reach levels of hepatocytes. Key TF of two of these clusters include SOX11, FOXQ1, and YBX3. The third unsuccessful cluster, controlled by HNF1, CAR, FXR, and PXR, strongly overlaps with genes repressed in cultivated hepatocytes compared to freshly isolated hepatocytes, suggesting that current *in vitro* conditions lack stimuli required to maintain gene expression in hepatocytes, which consequently also explains a corresponding deficiency of HLC.

**Conclusions:**

The present gene regulatory network approach identifies key transcription factors which require modulation to improve HLC differentiation.

## Graphical abstract

**Figure:**
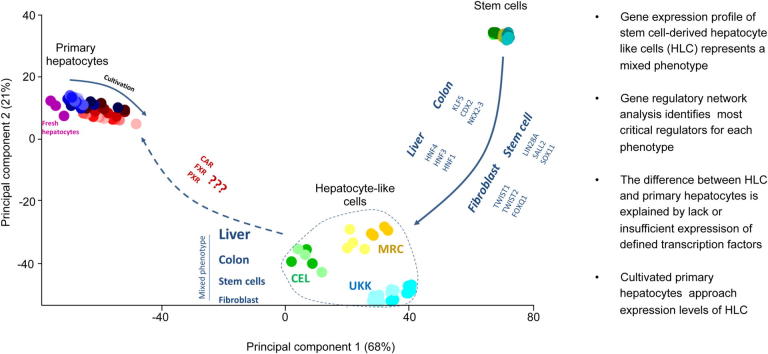

## Introduction

Primary human hepatocytes represent a well-established tool in pharmacology and toxicology [1] and have been applied for transplantation in metabolic liver diseases [2]. However, a major limitation is availability, since primary hepatocytes have to be isolated from surgically resected liver tissue [1]. Recently, protocols have been established and optimized to generate hepatocyte-like cells (HLC) from human embryonic stem cells (ESC) or human-induced pluripotent stem cells (hiPSC) [3], [4], [5], [6], [7], and human-induced pluripotent stem cells (hiPSC) [8], [9]. These protocols first differentiate the ESC or hiPSC into endodermal cells that are then further differentiated into hepatoblasts and HLC. These HLC express markers and functions of mature hepatocytes such as albumin (ALB) and transthyretin (TTR), and urea synthesis [7], [4], [9]. HLC present advantages over primary hepatocytes in terms of low batch-to-batch variation and, in principle, unlimited supply.

A limitation of the currently available characterizations of HLC derived from ESC or hiPSC is that they are usually based on a selected set of markers [10]. Furthermore, little is known about the transcriptional regulatory networks controlling the differentiation program. With the rationale that identification of suboptimal gene networks represents a tractable target for improving cell phenotype, we performed a whole genome gene array study, including the starting ESC or hiPSC populations from three different research centers, as well as the correspondingly differentiated HLC, and compared them to freshly isolated primary human hepatocytes. Two approaches were used to dissect the gene regulatory networks (GRN) controlling successful and undesired outcomes. First, we used the novel CellNet platform to determine the state and identity of differentiation in HLC, and to estimate control mechanisms by transcription factors (TF) represented by “network influence scores” [11]. Second, we generated gene clusters based on common expression patterns, and identified transcriptional regulators (i.e. TF) associated with each cluster. In addition, we showed a high correlation between genes with minimal upregulation in HLC and genes downregulated during cultivation of primary human hepatocytes, suggesting that the microenvironment of current culture systems is partly responsible for the insufficient differentiation of HLC.

In summary, we present evidence, based on unbiased bioinformatic analyzes, that HLC derived from ESC and hiPSC represent a mixed cell population and/or an intermediate cell type with features of liver, ESC, colon or fibroblasts. Moreover, we define a transcriptional regulatory framework that can be used for development of mature and homogeneous hepatocyte populations in the future.

## Materials and methods

### Human ESC cultivation and differentiation into HLC

**Fig. 1:**
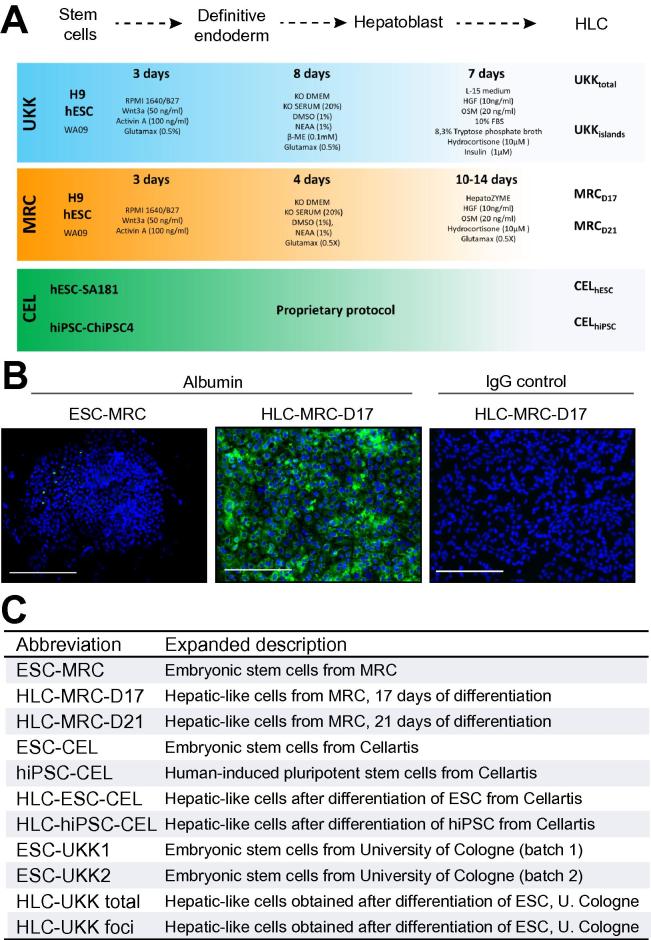
**Overview of stem cell differentiation protocols and gain of albumin expression in HLC.** (A) Schematic representation of cultivation conditions of stem cells to achieve a HLC phenotype in the three research centers involved in this study. (B) Fluorescent microscopy imaging of ESC and differentiated HLC from MRC stained with antibodies against albumin. The expression of albumin (green) can be detected uniformly in HLC after 17 days of differentiation. The specificity of the staining is confirmed by the absence of green color in HLC stained with IgG control antibodies. Nuclei are stained with DAPI (blue). Scale bars correspond to 100 μm. (C) Description of abbreviations and expanded description of all ESC and HLC models used in the current study.

For the present study, HLC were available from three different centers: University Klinik Köln, Germany (UKK), Medical Research Council Centre for Regenerative Medicine, Edinburgh UK (MRC) and Cellartis, Gothenburg, Sweden (CEL). The human ESC H9 (WA9, Wicell research institute, Madison; USA) (used by UKK and MRC) were cultured and propagated as described [7]. Cellartis used the commercial hESC and hiPSC cell lines SA181 and ChiPS4, for the generation of HLC CEL_hESC_ and CEL_hiPSC_ respectively [9]. HLC generated by MRC were collected after 17 (MRC_D17_) and 21 (MRC_D21_) days of differentiation [7]. HLC generated by the UKK protocol were collected after 18 days of differentiation. Since UKḰs protocol yields a mixed population of HLC islands and non-HLC, they were harvested as either as total (UKK_total_) population or as HLC foci (UKK_foci_). At least three independent experiments (biological replicas) were analyzed for all systems. Detailed descriptions of the protocols can be found in the Supplementary section (Supplementary Table 1). A schematic representation of the different cultivation protocols can be found in Fig. 1A.

### Primary human hepatocyte isolation and culture

Primary human hepatocytes were obtained under patient informed consent from surgical liver resection, following the 1975 Declaration of Helsinki as previously described [1]. Detailed protocols for isolation and culture of human hepatocytes in monolayer and sandwich systems are described in Godoy *et al.* 2013 [1] and in the Supplementary section.

### Microarray analysis

Analysis of gene expression in ESC, HLC and primary hepatocytes was performed with Affymetrix GenChip® Human Genome HG-U133 plus 2.0 chips (Santa Clara, CA, USA) as previously described [12], [13]. Gene expression levels in ESC, HLC and cultivated primary hepatocytes in collagen monolayer (CM) or collagen sandwich (CS) were compared to freshly isolated primary human hepatocytes (FH). Genes with a fold change greater than two over FH expression levels (*p* value <0.05, FDR corrected) were taken as significantly deregulated (Supplementary Table 2 for ESC and HLC; Supplementary Tables 3 and 4 for primary hepatocytes in monolayer or sandwich cultures respectively).

### Bioinformatics

The CellNet platform [11] was used to determine tissue identity based on gene expression profiles of ESC, HLC, and FH. The CellNet algorithm also generates a metric for GRNs associated with the genes belonging to specific tissue identities. The fuzzy c-means algorithm [14] was applied to generate gene clusters with similar expression patterns in ESC and HLC. Of the twenty clusters identified by this approach, we selected those with strongest changes in gene expression, and created five cluster groups, containing clusters with similar expression patterns (see Supplementary section for details). Enrichment analysis of gene ontology annotation was performed on the gene lists corresponding to each cluster group, using the manually curated gene ontology database of BioBase Knowledge Library (BKL) on the ExPlain™ web service (BioBase GmbH, Wolfenbüttel, Germany). Overrepresented TF binding sites on the promoters of genes in cluster groups were identified using the PRIMA (Promoter Integration in Microarray Analysis) algorithm [15] of the Expander Software 6.1 (EXPression ANalyzer and DisplayER [16].

Additional information on materials and methods including RNA isolation, cDNA synthesis, qRT-PCR, immunostaining and fluorescent microscopy are provided in the Supplementary section.

## Results

### Genome-wide characterization of ESC-derived hepatocyte-like cells

Human ESC-derived HLC were provided by three different centers (UKK, MRC, CEL) that focus on developing liver technologies from hESC and hiPSC. While the goal is the same, the experimental details and some differentiation factors differ between the protocols used by the three centers (Fig. 1A). The phenotype of HLC obtained by these protocols has already been published [3], [7], [9], [17], [31], [32]. Here, we confirmed the successful hepatic differentiation of HLC by immunofluorescence analysis of albumin, showing a relatively uniform expression in HLC populations (Fig. 1B).

**Fig. 2:**
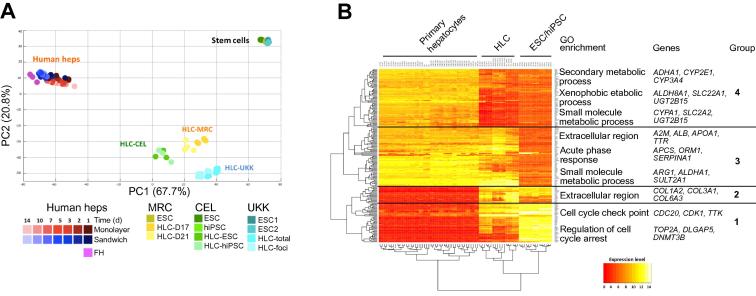
**Partial gain of hepatic differentiation in HLC.** (A) Principal component analysis generated with the 1000 genes with highest variance in stem cells and HLC from the three centers. The top two principal components represent 88.5% of the variance. The graph includes cultivated primary hepatocytes in 2D (monolayer) or 3D (sandwich) cultures. (B) Heat map representation of the 200 DEG with highest variance in ESC, HLC and primary hepatocytes (FH and cultivated in monolayer or sandwich). Representative GO terms and selected genes are indicated for each gene group.

To obtain an unbiased assessment of HLC differentiation, we performed whole genome gene array analysis of ESC as well as HLC and compared them to freshly isolated primary human hepatocytes. Cells were harvested as undifferentiated stem cells (ESC or hiPSC) or corresponding HLC (scheme for differentiation protocols, Fig. 1A; sample overview: Supplementary Table 1). Since the UKK differentiation method yields a mixed cell population with and without hepatocyte-like features, we harvested both whole cell preparations and HLC foci (Supplementary Fig. 1). A set of 10,420 differentially expressed genes (DEG) was defined for further evaluation, which were either differential between FH and HLC or between FH and ESC or hiPSC (*p* value <0.05; 2-fold threshold, FDR adjusted; Supplementary Table 2). Principal component analysis (PCA) of the 1000 genes with highest variance revealed the following features (Fig. 2A): (1) ESC (and hiPSC) approach primary hepatocytes after differentiation into HLC, however, they do not fully reach their position suggesting a partial gain of mature hepatic features; (2) although the HLC from the three centers result in distinct clusters, their overall position in relation to ESC and FH is close to each other, suggesting that the three protocols induce similar differentiation features. Nonetheless, HLC from MRC and CEL were closer to FH than HLC from UKK. This is also supported by Euclidean distance (ED) analysis (Supplementary Fig. 2; Supplementary Table 5); (3) since cultivation of primary hepatocytes induces dedifferentiation associated with massive deregulation of gene expression [1], we analyzed the gene expression profile of primary human hepatocytes cultivated for up to 14 days in sandwich and monolayer conditions [1]. Interestingly, overall gene expression values of the primary hepatocytes began to approach stem cell-derived HLC during the extended period of cultivation (Fig. 2A; EDs in Supplementary Fig. 3; Supplementary Table 6); (4) the ESC and hiPSC, although from different sources and cultivated in independent labs, cluster closely together. Similarly, the primary freshly isolated human hepatocytes, although from different donors, also adopt closely neighboring positions in the PCA; (5) modifications of the final differentiation steps, such as extending the incubation period from 17 to 21 days in MRC and the selective collection of hepatocyte-like foci in UKK had a relatively small influence on overall gene expression patterns. The partial hepatic differentiation of HLC was also evidenced by heat map representation and unsupervised clustering of the 200 genes with highest variance, followed by gene ontology enrichment analysis (Fig. 2B). For example, some genes associated with small molecule metabolic process were induced in HLC to levels comparable to primary hepatocytes (group 3), while many genes involved in xenobiotic metabolism remained low (group 4) (Fig. 2B). Likewise, cell cycle-associated genes were only partially repressed in HLC (group 1), and several extracellular matrix genes such as collagen 1A2 remained high in HLC compared to primary hepatocytes. In conclusion, HLC from the three centers were incompletely differentiated when compared to freshly isolated hepatocytes.

### GRN analysis demonstrates multi-organ differentiation in HLC

**Fig. 3:**
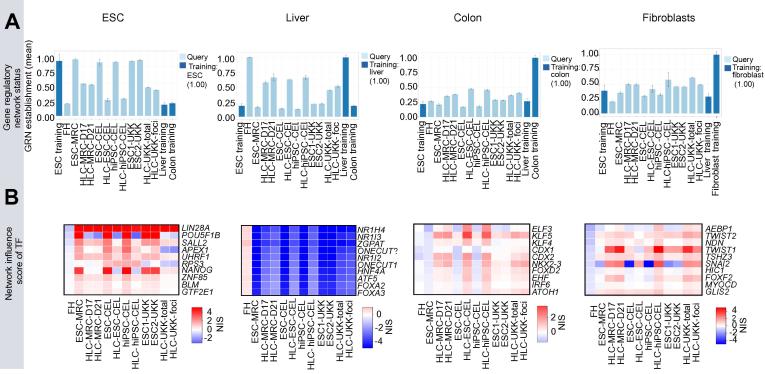
**CellNet allows identification of multi-tissue GRNs in HLC.** (A) Gene regulatory network (GRN) status in FH, ESC and HLC. Bars represent the strength of tissue-specific GRN. Each network (i.e. ESC, liver, colon and fibroblast) was established by a training set in CellNet, representing the maximum value of 1.0. The “ESC” GRN status is strongly repressed in all HLC compared with their corresponding ESC. Conversely, a gain of “liver” GRN is observed for all HLC cohorts. The “colon” GRN scores are also induced in all HLC, albeit to lower values than those of “liver” GRN. (B) Identification of transcription factors with highest influence over the ESC, liver and colon GRN. The CellNet algorithm establishes a metric for the influence of TF over the genes contained in each tissue GRN. The heat maps indicate the top ten TF with the highest influence on each GRN.

To further characterize these HLC, we applied a recently established bioinformatics algorithm (CellNet [11]), which assesses cell identity based on gene expression profiles and GRNs [11]. While FH and ESC scored a single tissue classification of “liver” and “esc” (respectively), HLC showed a mixed classification including “liver”, “esc”, “colon” and “fibroblast” (Supplementary Fig. 4). CellNet also estimates a metric of the GRN associated with the tissue classifications. The metric integrates TF influence over gene expression, based on experimental approaches including ChIP-Seq and gene expression profiles after TF overexpression or siRNA-mediated knockdown [11]). ESC and FH scored with a maximal GRN status (1.0) for “stem cell” and “liver”, respectively (Fig. 3A). All HLC showed a strong but not complete decrease in the “esc” GRN status, indicating that their stem cell features are not fully extinguished (Fig. 3A). Conversely, a strong increase in “liver” GRN was detected in all HLC compared with their corresponding ESC (Fig. 3A). Importantly, the “liver” GRN status in all HLC was higher than in the previously reported iHeps, reaching scores equal or higher than 0.5 in HLC (Fig. 3A), whereas iHeps only achieved 0.3 [18]. The GRN status also revealed an increase in “colon” and “fibroblast” status for all HLC (Fig. 3A), in agreement with the tissue classification analysis.

**Fig. 4:**
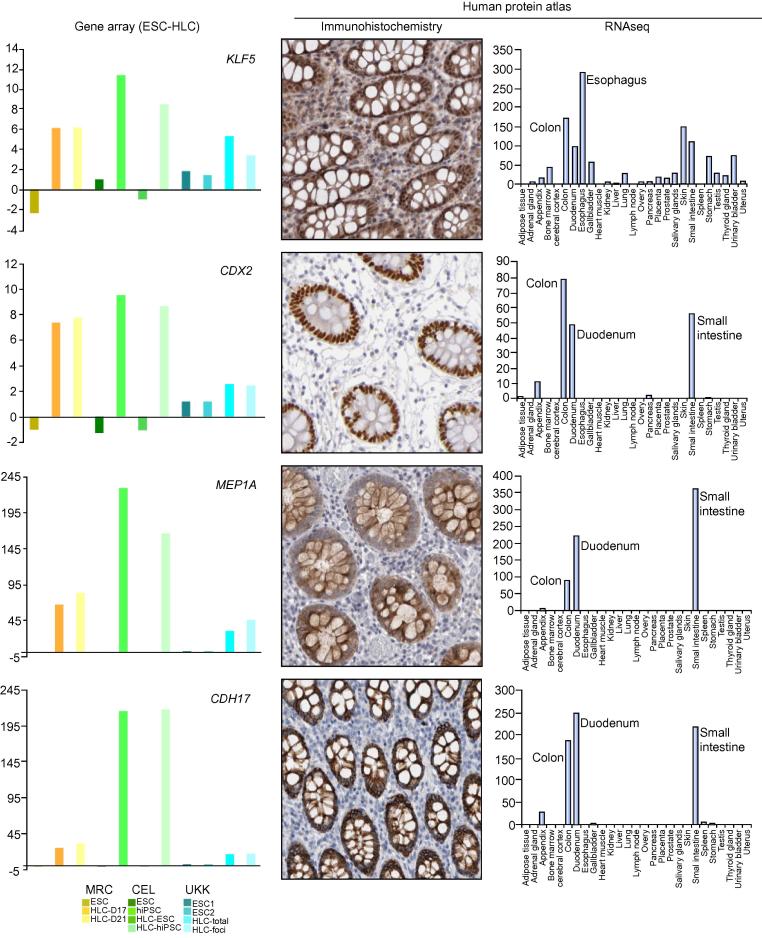
**Identification of colon-associated genes in HLC.** The graphs on the left indicate the mRNA expression levels of the transcription factors *KLF5* and *CDX2*, and the colon genes *MEP1A* and *CDH17*, in ESC and HLC. The tissue specificity for these genes was validated by querying the Proteinatlas® [30]. Here, representative pictures of immunostainings for the aforementioned genes are shown, indicating the nuclear expression of *KLF5* and *CDX2* in colon crypts, and the membranous expression of *MEP1A* and *CDH17*. The colon-enriched expression for these genes is also shown at the transcriptional level (RNAseq).

CellNet also generates a “network influence score” (NIS) whereby the potential influence of individual TF on each GRN is estimated [11]. The ESC network contains TF with known roles in stem cells such as *NANOG* and *POU5F1B*. The NIS of these TF was high in ESC and strongly reduced in HLC (Fig. 3B). Nonetheless, other TF such as *SALL2* and *LIN28A* remained with a high NIS in HLC (Fig. 3B). The NIS for the “liver” GRN identified several liver-enriched TF such as FXR (*NR1H4*), CAR (*NR1I3*), PXR (*NR1I2*), and *HNF4*, whose low influence in ESC was partially increased in all HLC (Fig. 3B). Likewise, NIS analysis identified several colon-enriched TF such as *KLF5*, *CDX2*, and *NKX2-3*, whose influence increased in HLC compared with their corresponding ESC (Fig. 3B). The expression of *KLF5* and *CDX2* were transcriptionally increased in HLC compared to FH (Fig. 4), supporting the estimation of their activity in HLC. Furthermore, several colon-enriched genes such as *MEP1A* and *CDH17* were also strongly induced in HLC (Fig. 4). The NIS of fibroblast-enriched genes contains genes such as *TWIST1* and *SNAIL2*, known to promote epithelial to mesenchymal transition (EMT), thereby representing an “unwanted” feature. Altogether, CellNet unveiled details of the differentiation stage of HLC, namely the acquisition of a mixed phenotype with partial loss of stem cell features and gain of hepatocyte, colon and fibroblast lineages.

### Cluster groups identify transcriptional regulators between successful and insufficient differentiation processes

**Fig. 5:**
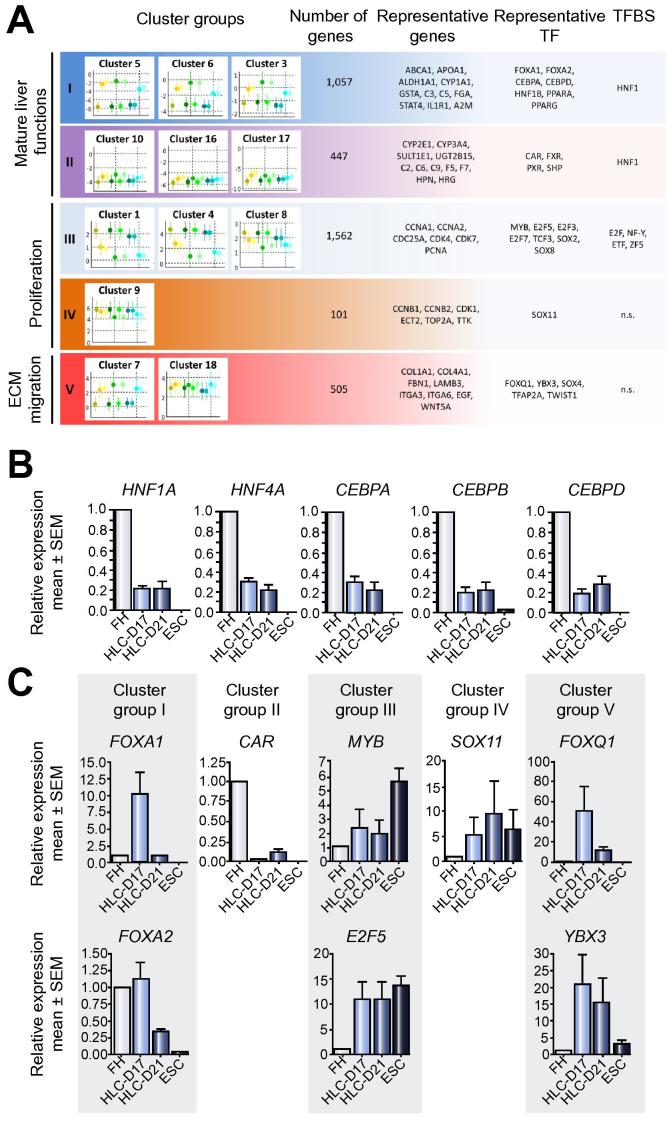
**Cluster groups unveil detailed features of complete and partial hepatic differentiation of HLC.** (A) After establishing 20 fuzzy clusters representing distinct gene expression patterns on HLC (see Supplementary section), five cluster groups were formed selecting clusters containing genes with average fold changes over five fold (see Supplementary section for detailed descriptions). The Cluster groups contain 3217 genes, allowing for a more robust and precise bioinformatics analysis. The most relevant biological motifs and representative genes are shown. Furthermore, the potential transcriptional control mechanisms such as deregulated (representative) transcription factors and overrepresented TFBS are shown. (B–C) Real time quantitative PCR analysis of selected transcription factors in ESC and HLC-MRC at 17 and 21 days of differentiation. Expression levels were normalized to those in freshly isolated hepatocytes. Bars correspond to mean values ± standard error of the mean. Representative of three independent biological replicas.

To further dissect transcriptional mechanisms controlling gene expression in HLC, we determined differentially expressed TFs and overrepresentation of TF binding sites (TFBS) in correlated gene groups. Twenty clusters were formed based on fuzzy c-means clustering (Supplementary Figs. 6 and 7; Supplementary Table 6; Supplementary materials and methods) [14] that were assigned to five cluster groups (Fig. 5A) (see Supplementary section).

Cluster group I contains 1057 genes with low expression in ESC that increased after the HLC differentiation protocols to levels comparable to those in primary hepatocytes. Conversely, cluster group II contains 447 genes with low expression in ESC that were minimally induced after differentiation into HLC (Fig. 5A). Cluster groups I and II are enriched in GO categories associated with mature liver functions, including “metabolic process” (GO:00081), “catalytic activity” (GO:00038) and “immune system process” (GO:00023) (Fig. 5A, representative genes in Supplementary Table 8). Hence, cluster group I represents successful hepatocyte differentiation of HLC, while the genes in cluster group II did not reach the levels of hepatocytes. Cluster group I contains several transcription factors with known roles in hepatocyte differentiation [19], including transcriptionally upregulated *HNF1A*, *HNF4A*, *CEBPA*, *CEBPD*, and *CEBPD* (Fig. 5A, Supplementary Table 9). The upregulation of these TF was confirmed by quantitative real time PCR (Fig. 5B). Furthermore, the binding site for HNF1 was significantly overrepresented in cluster I (Fig. 5A; Supplementary Table 10). The increased expression of these TF is consistent with the successful hepatocyte differentiation of HLC, since their role as inducers of liver-specific genes is well-established [19]. Cluster group II also contains TF controlling liver metabolic functions, such as *CAR*, *FXR*, *SHP*, *PXR* (Fig. 5A and Supplementary Table 9). In contrast with TF of cluster group I, these TF were only slightly induced in HLC (Fig. 5A, B; Supplementary Table 9). The low expression of these TF in HLC is consistent with the NIS results for the “liver” GRN. The binding site for HNF1 was also overrepresented in cluster group II. The complexity of the GRNs is illustrated by the fact that overrepresented binding sites of HNF1 appear in both, cluster I and II. The difference between both clusters may be due to HNF1 interaction partners. Notably, *FXR* and *PXR*, known to functionally interact with HNF1, also show low expression levels in HLC (Fig. 5 and Supplementary Table 9).

Cluster group III contains 1562 genes whose expression was high in ESC and strongly decreased during differentiation into HLC (Fig. 5A). Conversely, cluster group IV contains 101 genes with high expression levels in ESC that did not decrease to the levels of FH during the differentiation process (Fig. 5). Cluster group III is enriched in GO terms such as “cell cycle” (GO:0007049) and “regulation of mitosis” (GO:0007088) (Supplementary Table 8). Genes in these categories include cyclins as well as cyclin-dependent kinases, and TF and TFBS, such as E2F5 and MYB (Fig. 5; Supplementary Tables 9–10). The downregulation of cell cycle-associated genes suggests repression of the self-renewal potential, a well-known feature of stem cell differentiation. Cluster IV also contains GO terms associated to cell cycle such as “regulation of nuclear division” and “regulation of mitosis”, and included genes such as *CCNB1*, *CDK1*, *ECT2*, *TOP2A*, and *TTK*. Although the expression of these genes was downregulated in HLC compared to stem cells, their levels remained above those of primary hepatocytes (Fig. 5A; Supplementary Table 8). It contains only one TF, namely *SOX11* (Supplementary Table 9). The observation that cell cycle-associated genes were not fully downregulated in HLC prompted us to determine the expression levels of the representative TF identified by our analysis by real time qPCR (Fig. 5C). While *MYB* expression was strongly reduced in HLC, the levels of *E2F5* and *SOX11* remained elevated (Fig. 5C), suggesting that they may be responsible for the sustained expression of cell cycle-associated genes in cluster group IV.

Cluster group V contains genes whose expression is at least 4-fold higher in stem cells compared to hepatocytes and which further increase during differentiation. This cluster shows overrepresentation of GO terms such as “extracellular region” (GO:0005576) and “cell migration” (GO:0016477) (Fig 5A; Supplementary Table 8). Genes in these categories include extracellular matrix genes such as collagens (e.g. *COL1A1*, *COL12A1*), other matrix proteins (FBN1, LAMB3, LAMA5), integrins (e.g. ITGA3, ITGA6, ITGAV) and cytokines (EGF, WNT5A). The deregulated TF identified in this cluster group, including *FOXQ1*, *YBX3*, *TWIST1*, and *SOX4*, may be responsible for the not fully differentiated phenotype of HLC (Fig. 5 and Supplementary Table 9). For example, *FOXQ1* is an oncogene found overexpressed in colorectal [20] and breast cancer [21], where it induces proliferation, dedifferentiation and EMT. *FOXQ1* is also overexpressed in hepatocellular carcinoma and induces metastasis [22]. Similarly, *YBX3* can induce proliferation and dedifferentiation [23], [24]. The upregulation of these TF in HLC was also demonstrated by real time qPCR analyzes (Fig. 5C). Therefore, they represent an undesirable outcome of the differentiation process.

In conclusion, cluster group analysis reveals gene groups representing successful and failed outcomes in HLC differentiation. Furthermore, we identify key TF responsible for aberrant expression clusters, thereby giving a basis for overexpression or knockdown approaches to improve HLC differentiation.

### Culture-dependent repression of liver metabolism genes

**Fig. 6:**
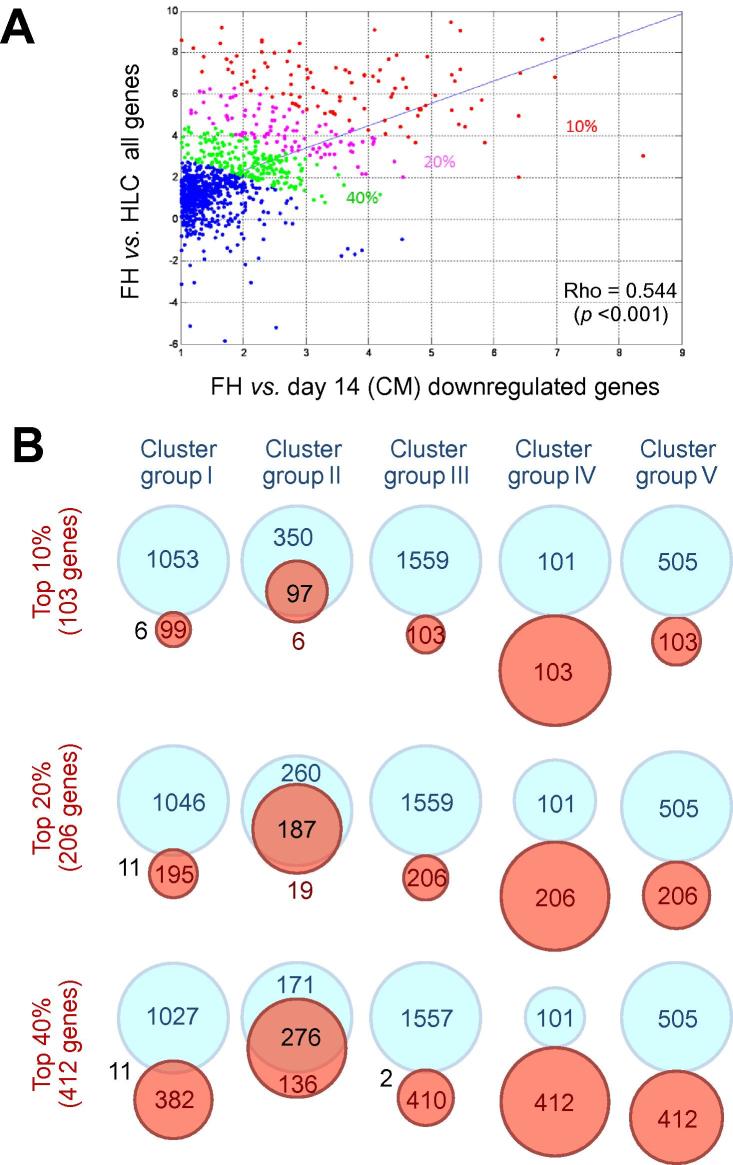
**Comparable repression of metabolism-associated genes during cultivation of primary hepatocytes and differentiated HLC.** (A) Correlation between genes repressed in primary hepatocytes after 14 days of cultivation in 2D (monolayer) conditions and genes deregulated in HLC (UKK-islands) compared to FH. The red dots represent the top 10%, while the purple and green dots correspond to the additional 20 and 40% genes with highest correlation. (B) Venn diagram analysis indicates the high overlap between top 10, 20, and 40% correlated genes with genes in cluster group II (Fig. 5). The light blue circles represent genes in each cluster group as described in Fig. 5. Red circles represent genes in the top 10 to 40% with highest correlation, as described in A.

It is well-known that primary hepatocytes dedifferentiate in culture, whereby expression of many metabolic genes decreases. Interestingly, *in vitro* dedifferentiated primary hepatocytes resemble stem cell-derived HLC more closely than freshly isolated hepatocytes, which can be seen from the PCA (Fig. 2A) and can also be objectified by the analysis of EDs (Supplementary Fig. 3; Supplementary Table 6). Therefore, one might hypothesize that if the same genes which decrease during cultivation in primary hepatocytes, also remain low after differentiation of stem cells into HLC, this is due to the the creation of a sub-optimal liver niche in cell culture. To test this hypothesis, we analyzed the correlation between downregulated genes after 14 days of monolayer culture of primary hepatocytes and the difference between HLC *vs.* freshly isolated hepatocytes (gene expression in HLC minus expression in freshly isolated hepatocytes) (Fig. 6A). Interestingly, a highly significant correlation was obtained (*p* <0.001; R = 0.54). We further analyzed the 10, 20, and 40% genes with the highest correlation coefficients, indicated by red, purple and green colors respectively in Fig. 6A. Interestingly, these genes belong predominantly to the aforementioned cluster group II (Fig. 6B). This correlation remained even when including the top 20 or 40% genes (Fig. 6B). As described above, metabolism-associated GO terms are overrepresented in this cluster group. Similarly, metabolism GO terms were enriched in the top 10 to 40% correlated genes (Supplementary Table 11). All these metabolism-associated genes are expressed at much lower levels in HLC than in primary hepatocytes, independent of which of the three differentiation protocols (MRC, CEL, UKK) the HLC were obtained (Supplementary Table 12). The same genes were also downregulated during a 14 days cultivation period of primary hepatocytes, when the hepatocytes were kept in 2D monolayer cultures (Supplementary Table 12). To see if decreased expression of metabolic genes is explained by the culture conditions we also used a 3D culture system, where hepatocytes were cultivated between two layers of soft gel collagen (Supplementary Table 12). 3D culture ameliorated or abolished the decrease in expression for some genes including *ADH1C*, *CYP4F2*, *CYP7A1*, and *HSD11B1*, but the situation remained similar for the majority of genes. In conclusion, mechanisms inherent to *in vitro* cultivation systems antagonize the differentiation towards mature liver, affecting both the stability of primary hepatocytes and the potential for gain of liver functions in HLC.

## Discussion

The differentiation of human embryonic stem cells (hESC) to HLC and the advent of induced pluripotency seem to open exciting new possibilities to generate an unlimited supply of human hepatocytes for numerous applications [2], [25]. Indeed, recent studies reported that pluripotent stem cell derived HLC and transdifferentiated iHep ‘exhibited hepatic functions’ comparable to cryopreserved human hepatocytes [7], [4], [10]. However, also more critical viewpoints about the differentiation status of HLC have been published [26], [18]. The present genome-wide study demonstrates that the interpretation of stem cell derived or transdifferentiated-derived HLC as almost fully differentiated hepatocytes might be further away than previously suggested. In reality, a mixed population of cells are produced which express genes representative of liver, colon, stem cells and/or fibroblasts. This seems to be in agreement with principles of embryonic development. After gastrulation, the naive endoderm transforms into a primitive gut tube which becomes regionalized into foregut, midgut, and hindgut domains. As development proceeds, the foregut gives rise to the liver, while the hindgut forms the large intestine. In line with this, a recent proteomics approach demonstrated that HLC resemble embryonic rather than mature liver [27]. Results of the present GRN analysis show that HLC represent a population of cells that possess mixed features with high expression of colon-associated TF such as KLF5, CDX2, and NKX2-3 (Supplementary Fig. 12). To further differentiate HLC to mature hepatocytes it will be useful to understand the cell populations which exist in vitro and modulate transcription factors responsible for colon development. However, care must be taken with this approach as colon transcription factors, such as CDX2, also modulate HNF4a DNA occupancy and gene expression [33].

A strength of the present study is that GRN analysis of HLC established by three different labs resulted in very similar conclusions. Moreover, cluster analysis demonstrated that gene expression alterations during the differentiation process fall into five distinct groups. Cluster group I supports the ‘favorable’ interpretation obtained in several previous studies. It contains genes strongly upregulated during the differentiation process that are involved in hepatic metabolism (Supplementary Table 11). However, it should be considered that also cluster group II contains genes representative of normal liver functions, such as metabolism and synthesis of coagulation and complement factors. In contrast to cluster I they remain orders of magnitudes lower than those in primary hepatocytes. Interestingly, cluster II genes largely overlap with genes whose expression decreases during cultivation of primary hepatocytes (Fig. 5). This striking overlap could mean that key mechanisms responsible for maintenance of the liver’s metabolic genes are generally absent *in vitro*. Discovery of factors that maintain expression of cluster IV genes in primary hepatocytes may also help to improve stem cell differentiation. The binding site of HNF1, a TF involved in expression of genes characteristic of differentiated hepatocytes [19] is overrepresented in cluster group II genes; further ‘liver transcription factors’, such as CAR, FXR, PXR are upregulated during stem cell differentiation but only to levels much lower than those observed in primary hepatocytes.

The second ‘favorable’ cluster group III contains proliferation associated genes that are suppressed during the differentiation process, thereby approaching the low expression levels of primary hepatocytes. In contrast, cluster group IV contains also proliferation associated genes, but expression levels stay higher compared to primary hepatocytes. *SOX11* seems to represent a critical transcription factor for these genes. Cluster group V contains genes whose expression is upregulated in comparison to levels in mature hepatocytes. These genes include extracellular matrix proteins and integrins as well as the TF *TWIST1* which previously have been reported to be upregulated when primary hepatocytes dedifferentiate in culture [28], [29], [13]. These “unfavorable” clusters (II, IV and V) contain only 1053 genes (28.7%) compared to the much higher number of 2619 genes (71.3%) of the favorable clusters (I and III). Therefore, the majority of genes whose expression is influenced during differentiation approach levels similar to those observed in hepatocytes. Nevertheless, a major hurdle on the path to stem cell-derived hepatocytes are control mechanisms of gene cluster groups II, IV and V. Since the transcription factors controlling their expression have been identified in this study, research in the future should aim at adjusting their activity to levels of primary hepatocytes.

In addition to hESC-derived HLC, three independent batches of hiPSC-HLC were analyzed. Bioinformatic analyzes demonstrate a high degree of similarity to hESC-HLC considering the position in the PCA and cluster analysis. A minor difference is that the proliferation associated cluster group III genes were lower in hiPSC-HLC compared to hESC-HLC. Differences between hESC-HLC populations were also noted, with the stronger suppression of ESC gene network features in CEL-HLC compared to those from UKK and MRC (Fig. 3). While promising, the CEL protocol also resulted in enhanced colon network features when compared to the other HLC. This illustrates that more efficient suppression of ESC features does not necessarily lead to enhanced hepatocyte but may enhance the “unwanted” colon features.

In conclusion, the present unbiased, genome-wide study reveals the strengths and limitations of current protocols aiming at generating hepatocytes from hESC or hiPSC. The observation of mixed cell specification, the definition of five cluster groups, and their assignment to key TF give a basis to radically improve future differentiation protocols.

## Financial support

This study was supported by the European Union Seventh Framework Programme (FP7)-Health projects DETECTIVE (EU-project FP7-Health Grant Agreement No. 266838) and NOTOX (EU-project FP7-Health Grant Agreement No. 267038), and the BMBF (German Federal Ministry of Education and Research) project Virtual Liver (0313854). Dr David C. Hay was supported by the UK Regenerative medicine platform (MR/K026666/1 and MR/L022974/1). Mr. Baltasar Lucendo-Villarin and Ms. Dagmara Szkolnicka were funded by MRC PhD studentships.

## Conflict of interest

The authors who have taken part in this study declared that they do not have any conflict of interest with respect to this manuscript.

## Author’s contributions

PG, DCH, JGH were responsible for the overall conception design of the study. WSH, AS, JS, performed gene array analysis and bioinformatics analyzes. TSW, GD, AN assisted with the acquisition of fresh primary human hepatocytes. AW, RS, GC, SH performed the cultivation of primary human hepatocytes. DS and BLV conducted experiments to obtain HLC-MRC. BKM, PB, and AA conducted experiments to obtain HLC-CEL. KN, AS HLC-UKK conducted experiments to obtain HLC-UKK. PG, JGH, DH, KE, PB, BKM, AN all contributed to the interpretation of data, drafting of article and critical revision of the manuscript.
