# Corrigendum to “Gene networks and transcription factor motifs defining the differentiation of human stem cells into hepatocyte-like cells” [J Hepatol 2015;63:934–942]

**DOI:** 10.1016/j.jhep.2015.11.003

**Published:** 2016-02

**Authors:** Patricio Godoy, Wolfgang Schmidt-Heck, Karthick Natarajan, Baltasar Lucendo-Villarin, Dagmara Szkolnicka, Annika Asplund, Petter Björquist, Agata Widera, Regina Stöber, Gisela Campos, Seddik Hammad, Agapios Sachinidis, Umesh Chaudhari, Georg Damm, Thomas S. Weiss, Andreas Nüssler, Jane Synnergren, Karolina Edlund, Barbara Küppers-Munther, David C. Hay, Jan G. Hengstler

**Affiliations:** 1 IfADo-Leibniz Research Centre for Working Environment and Human Factors at the Technical University Dortmund, Dortmund, Germany; 2 Leibniz Institute for Natural Product Research and Infection Biology eV-Hans-Knöll Institute, Jena, Germany; 3 University of Cologne, Institute of Neurophysiology and Center for Molecular Medicine Cologne (CMMC), Robert-Koch-Str. 39, 50931 Cologne, Germany; 4 MRC Centre for Regenerative Medicine, University of Edinburgh, Edinburgh EH16 4UU, United Kingdom; 5 Takara Bio Europe AB (former Cellartis AB), Arvid Wallgrens Backe 20, 41346 Gothenburg, Sweden; 6 Systems Biology Research Center, School of Bioscience, University of Skövde, Sweden; 7 NovaHep AB, Arvid Wallgrens Backe 20, 41346 Gothenburg, Sweden; 8 Charité University Medicine Berlin, Department of General-, Visceral- and Transplantation Surgery, D13353 Berlin, Germany; 9 Center for Liver Cell Research, Department of Pediatrics and Juvenile Medicine, University of Regensburg Hospital, Regensburg, Germany; 10 Eberhard Karls University Tübingen, BG Trauma Center, Siegfried Weller Institut, D72076 Tübingen, Germany; 11 Department of Physiology, Faculty of Biological Sciences, University of Concepción, Chile

An error was introduced in Fig. 4 of the original manuscript. Please find the corrected figure below. The authors apologize for any inconvenience.

**Fig. 4:**
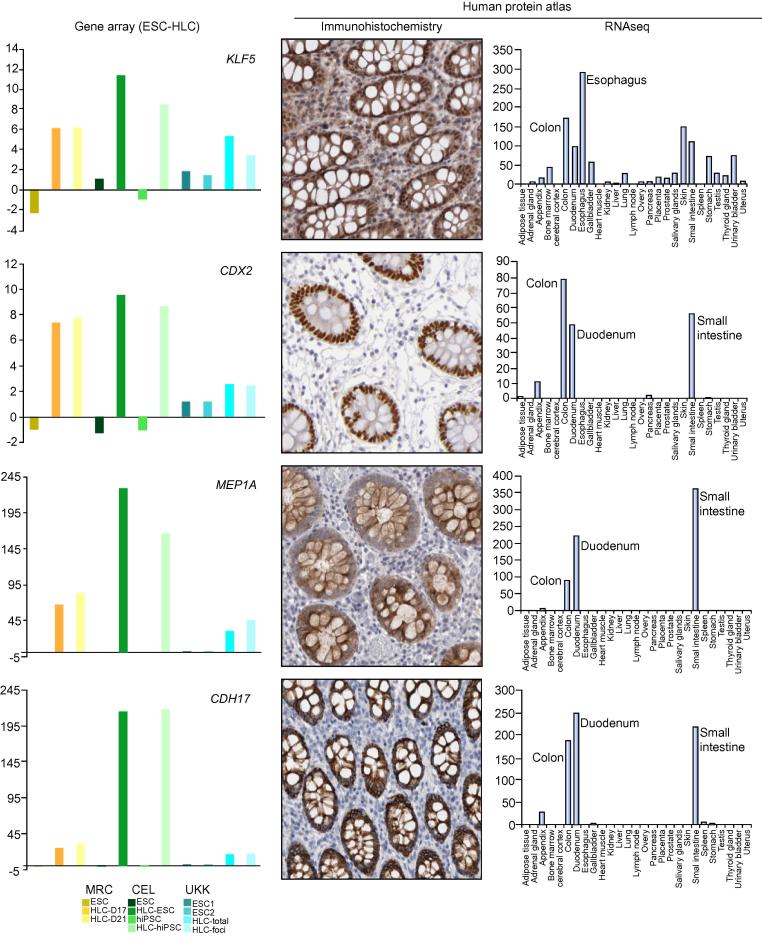
**Identification of colon-associated genes in HLC.** The graphs on the left indicate the mRNA expression levels of the transcription factors *KLF5* and *CDX2*, and the colon genes *MEP1A* and *CDH17*, in ESC and HLC. The tissue specificity for these genes was validated by querying the Proteinatlas® [30]. Here, representative pictures of immunostainings for the aforementioned genes are shown, indicating the nuclear expression of *KLF5* and *CDX2* in colon crypts, and the membranous expression of *MEP1A* and *CDH17*. The colon-enriched expression for these genes is also shown at the transcriptional level (RNAseq). (This figure appears in colour on the web.)

