# Supplementary material for: Gene networks and transcription factor motifs defining the differentiation of stem cells into hepatocyte-like cells
Source: J Hepatol. 2015 Oct;63(4):934–42. doi: 10.1016/j.jhep.2015.05.013 (PMC4580233; doi:10.1016/j.jhep.2015.05.013)
Supplement: Supplementary data [file mmc12.pdf]

Supplemental data to

Gene networks and transcription factor motifs defining the differentiation of stem cells into hepatocyte-like cells

Patricio Godoy, Wolfgang Schmidt-Heck, Karthick Natarajan, Baltasar Lucendo-Villarin, Dagmara Szkolnicka, Annika Asplund, Petter Bjorquist, Agata Widera, Regina Stoeber, Gisela Campos, Seddik Hammad, Agapios Sachinidis, Georg Damm , Thomas S. Weiss, Andreas Nussler, Jane Synnergren, Karolina Edlund, Barbara Küppers-Munther, David Hay, Jan G. Hengstler

Table of contents

Supplemental materials and methods

Human embryonic stem cell cultivation and differentiation into Hepatocyte-Like Cells
(HLC)……………………………………………………………………………………..4

Primary human hepatocyte isolation and culture……………………………………5

RNA isolation ……………………………………………………………………...6

Affymetrix® gene array analysis………………………………………………………7

Microarray processing and statistical analysis………………………………………7

Fuzzy clustering……………………………………………………………………..8

Cluster group definition……………………………………………………………..9

Gene set enrichment analysis (Gene ontology)……………………………………10

Transcription factor binding site (TFBS) enrichment analysis………………………10

Identification of deregulated transcription factors ……………………………………11

Euclidian distance analysis…………………..………………………………………11

CellNet analysis………………………………………………………………………12

cDNA synthesis and quantitative real time PCR ……………………………………12

Immunostaining and fluorescent microscopy ……………………………………...13

Supplemental figures……………………………………………………………….15

Supplemental table legends………………………………………………….……26

Ethics committee approval for patient informed consent form for donation of liver resections ……………………………………………………………………….31

Supplemental references………………………………………………………...33

Human embryonic stem cell cultivation and differentiation into Hepatic-Like Cells (HLC)

Differentiation into HLC was performed in three laboratories, namely University Klinikum Köln (UKK), Medical Research Council Centre for Regenerative Medicine (MRC) and Cellectis (CEL). For UKK, H9 cells were seeded on Matrigel (BD Biosciences, Heidelberg, Germany) coated dishes and allowed to grow until 60-70% confluence. Afterwards, the cells were washed twice with sterile PBS and further cultured in "***definitive endoderm differentiation medium***" RPMI 1640-Glutamax basal medium (Life Technologies, Karlsruhe, Germany) supplemented with 100 ng/ml Activin A (Peprotech, Hamburg, Germany), 50 ng/ml Wnt3a (R&D Systems, Wiesbaden-Nordenstadt, Germany) and B-27 (Life Technologies, Karlsruhe, Germany). After three days, differentiation medium was changed to ***"hepatoblast differentiation medium"*** consisting of Knock Out DMEM medium (Life Technologies, Karlsruhe, Germany), supplemented with 20% v/v knock out serum replacement medium (Life Technologies, Karlsruhe, Germany), 1% DMSO (Sigma, Munich, Germany), 1% non-essential amino acids (Life Technologies, Karlsruhe, Germany), 0.5% Glutamax (Life Technologies, Karlsruhe, Germany) and 0.1 mM β-mercaptoethanol (Life Technologies, Karlsruhe, Germany). After eight days, cells were changed to "***hepatic maturation medium***" consisting of L-15 basal medium (Sigma, Munich, Germany) supplemented with 10 ng/ml HGF (Peprotech, Hamburg, Germany), 10% FBS (Invitrogen), 20ng/ml Oncostatin M (R&D systems, Wiesbaden-Nordenstadt, Germany), 8.3% tryptose phosphate broth (Sigma, Munich, Germany), 10µm hydrocortisone-21-hemisuccinate (Sigma,

Munich, Germany) and 1 µM insulin (Sigma, Munich, Germany). At all differentiation steps the medium was refreshed daily. The cells were harvested for RNA analysis after 7 days in hepatic maturation medium.

MRC used a similar protocol as UKK with some modifications as described [1]. Briefly, hESC were grown at 30% confluency, washed twice with sterile PBS and cultured for 3 days in "***definitive endoderm differentiation medium***" as described for UKK, excluding the addition of Glutamax®. Afterwards, the cells were cultured for 4 days in "***Hepatoblast differentiation medium"*** with a similar composition as UKK, excluding β-mercaptoethanol. Finally, the cells were cultured for additional 10 to 14 days in "***hepatic maturation medium***" again with a similar composition as UKK, excluding FBS, tryptose phosphate, insulin and Glutamax®. During definitive endoderm specification the medium was changed daily. At the subsequent stages of hepatocyte differentiation the medium was changed every 48 hours. At all differentiation steps the medium was refreshed daily. The cells were harvested for RNA analysis after 10 and 14 days in hepatic maturation medium, corresponding to 17 and 21 days of differentiation, respectively [2].

CEL used the commercial hESC and hiPSC cell lines SA181 and ChiPS4, for the generation of hESC-Hep and hiPSC-Hep respectively [3] and used a company-owned, protected differentiation protocol.

Primary human hepatocyte isolation and culture

Primary human hepatocytes were obtained under patient informed consent from liver sections of patients undergoing surgical liver resection (following the 1975 Declaration of Helsinki) as previously described [4]. Detailed protocols for isolation and culture of human hepatocytes are described in Godoy et al 2013 [4]. Briefly, hepatocytes were obtained by two step collagenase-I perfusion, removed of debris by Percoll centrifugation, and plated in 6 well dishes either coated with collagen-I (collagen monolayer) or between two layers of collagen-I gel (collagen sandwich) at a density of 104,166 or 88,542 cells/cm^2 respectively. The cells were cultured in serum-free William's E medium (PAN Biotech, Aidenbach, Germany) for the indicated time. Medium was changed daily.

RNA isolation

Total RNA was isolated from stem cells, HLC and primary human hepatocytes using the Phenol/Chloroform method (Trizol®, Qiagen, Hilden, Germany) according to the manufacturer's description. For freshly isolated hepatocytes, 1 million cells in suspension medium were allowed to decant in a 1.5 ml conical tube placed on ice for 10 minutes. The medium was quickly removed and the cells homogenized in 1 ml of Trizol® (Qiagen, Hilden, Germany) by 10 repetitive aspirations in a micropipette. For primary hepatocyte, stem cells and HLC in culture, the medium was removed at the corresponding time point and the cells were homogenized in 1 ml Trizol® (Qiagen, Hilden, Germany) with a micropipette. RNA concentration and integrity and were determined spectrophotometrically in a Nanodrop®2000

(ThermoScientific, Waltham MA, USA) and in a Bioanalyzer® (Agilent, Waldbronn, Germany) respectively.

Affymetrix® gene array analysis

Affymetrix gene array analysis was performed as previously described [5] [6], using the Affymetrix GenChip® Human Genome HG-U133 plus 2.0 (Santa Clara, CA, USA). Briefly, five µg RNA were transcribed into cDNA by oligo dT primers, and reverse transcribed to biotinylated cRNA with the Gene Chip IVT® Labeling kit (Affymetrix, High Wycombe, UK). Cleanup of the IVT product was done using CHROMA SPIN-100 columns (Clontech, Saint-Germaine-en-Laye, France). Spectrophotometric analysis was used for quantification of cRNA with acceptable A260/A280 ratio of 1.9 to 2.1. After that, the cRNA was fragmented following Affymetrix's protocol. Labeled and fragmented cRNA was hybridized to Human Genome HG-U133 plus 2.0 Affymetrix GeneChips for 16h at 45° C according to the manufacturer's instructions. Microarrays were washed using an Affymetrix fluidics station 450 and stained initially with streptavidin-phycoerythrin. For each sample the signal was further enhanced by incubation with biotinylated goat anti-streptavidin followed by a second incubation with streptavidin-phycoerytherin and a second round of intensities were measured. Microarrays were scanned with an Affymetrix scanner controlled by Affymetrix Microarray Suite software.

Microarray processing and statistical analysis

Affymetrix gene expression data were pre-processed using ‘affyPLM’ packages [7] of the Bioconductor Software [8]. To obtain the genes with the strongest evidence of differential expression, a linear model fit was applied for each gene using ‘limma’ (Linear Models for Microarray Data) packages [9] of [8]. Data obtained from fresh hepatocytes were used as reference. The custom chip definition file from Brainarray [10] based on Unigene ID’s was used to annotate the microarrays. A false positive rate of a=0.05 with FDR correction and a fold change greater 2 was taken as the level of significance. A list with all differentially regulated genes (DEG) in stem cells and HLC can be found in **Suppl. Table 1**. The DEG in cultivated primary hepatocytes on collagen monolayer or collagen sandwich can be found in **Suppl. Table 2 and 3**, respectively. Processing and visualization (Principal Component Analysis) of data were performed using MATLAB tools (The MathWorks Inc., Natick, MA, USA).

Fuzzy clustering

The time-profiles of the differentially expressed genes in stem cells and in HLC were scaled between their respective absolute temporal extreme values to focus subsequent cluster analysis on the qualitative behavior of the expression profiles. The time series were clustered using fuzzy c-means [11] (fuzzy exponent = 1.5; maximum number of iteration = 200; minimum cost function improvement = 10-10). The optimum number N of cluster was estimated by repeated calculation (number

of iterations = 100) of the fuzzy cluster index ‘Separation Index’ (**Suppl. Fig. 6**). A list with genes belonging to each fuzzy cluster is provided in **Suppl. Table 6**.

Cluster group definition

The generation of gene clusters generated by fuzzy c-means revealed two features that allowed a more precise interpretation of the transcriptional changes induced by differentiation of ESC into HLC: the magnitude (i.e. fold) difference between ESC (or HLC) and FH, and the magnitude of changes induced by the differentiation into HLC (**Suppl. Fig. 7**). For example, cluster 5 contained genes with very low expression in ESC (i.e. FGB-fibrinogen beta chain; -1,011-fold in MRC-ESC v/s FH) which were strongly induced upon differentiation into HLC (1.03-fold in MRC-HLC_{D21} v/s FH), representing a desired outcome in HLC. Since similar features were observed in clusters 6 and 3, albeit with lower magnitudes in gene expression changes (**Suppl. Fig. 7**), we considered them as representing a common response. Conversely, cluster 17 consisted of genes with very low expression in ESC (i.e. CYP2E1; -808.3-fold in MRC-ESC v/s FH) that were minimally induced after differentiation into HLC (-761.12-fold in MRC-HLC_{D21} v/s FH), representing a failure in establishing a transcriptional profile similar to FH (**Suppl. Fig. 7**). Likewise, cluster 4 contained genes highly expressed in ESC (i.e. POU5F1P3; 43.48-fold in MRC-ESC v/s FH) that were strongly repressed in the corresponding HLC (-1.02-fold in MRC-HLC_{D21} v/s FH). Similar features were observed in clusters 1 and 8, albeit with lower magnitudes in gene expression changes (**Suppl. Fig. 7**),

representing successful downregulation of cell cycle processes in HLC. Conversely, cluster 9 consisted of highly expressed genes in ESC (i.e. GJA1-gap junction protein, alpha1; 537.24-fold in MRC-ESC v/s FH) that were minimally repressed after differentiation into HLC (292.12-fold in MRC-HLC$_{D21}$ v/s FH) (**Suppl. Fig. 7**), representing and undesired outcome. The analysis also revealed clusters consisting of genes with minimal differences in ESC v/s FH (i.e. SLC8A3-solute carrier family 8; -1.19-fold in MRC-ESC v/s FH) and whose expression were only slightly altered in HLC (-1.2-fold in MRC-HLC$_{D21}$ v/s FH) (**Suppl. Fig. 7**).

Gene set enrichment analysis (Gene ontology)

Genes which show change of the ratios higher (or lower) than 2-fold in the arrays at any of the time points have been considered as up or downregulated and subjected for gene ontology (GO) analyses. Differentially regulated genes of each Cluster group were categorized using the manually curated Gene Ontology of the BioBase Knowledge Library (BKL) of the ExPlain™ webservice (BioBase GmbH, Wolfenbüttel, Germany). The analysis was performed on gene lists corresponding to the combination of clusters into Cluster groups as described in the results section. The results are provided in **Suppl. Table 7**.

Transcription factor binding site (TFBS) enrichment analysis

To identify transcription factors (TFs) whose binding sites are enriched in a given set of promoters, the algorithm PRIMA (PRomoter Integration in Microarray Analysis [12]) of the Expander Software 6.1 (EXPression ANalyzer and DisplayER; [13]) was used. The analysis was performed on genes belonging to the each Cluster group that are significantly (p-value cut off is 0.05) changed more than 2-fold. All human genes (Ensembl release 42) were used as background set, the threshold of the p-value was set to 0.01 and the region was scanned from -3000 to +200. The results are provided in **Suppl. Table 9**.

Identification of deregulated transcription factors

To identify deregulated transcription factors (TFs) in HLC cluster groups, the genes were mapped to specific transcription factor using the database TRANSFAC Professional and classified using the ExPlain™ webservice. The results are provided in **Suppl. Table 8**.

Euclidian distance analysis

In order to evaluate the differentiation process, the Euclidean distance between the different cell types was calculated based on the expression level of differentially expressed genes**.** Genes with a fold change greater than 2 and a false discovery rate (FDR) adjusted p-value lower than 0.05 were considered as differentially expressed. The pairwise Euclidean distances were calculated by using the Statistic

toolbox of Matlab (Mathworks Inc., USA). The results are provided in **Suppl. Tables 4 and 5**.

CellNet analysis

To assess the fidelity of cell conversion a classification probability related to 16 specific tissues and cells was calculated. Furthermore a metric gene regulatory networks (GRN) status was calculated for further quantification of the established state of differentiation process [14]. A network influence score (NIS) was calculated for cell and tissues with a high value of the GRN status to estimate the importance of transcriptional regulators to normal and dysregulated GRNs. All calculations were done using the locally available R version of the software CellNetr http://pcahan1.github.io/cellnetr/**.**

cDNA synthesis and quantitative real time PCR

For complementary DNA synthesis, 2 µg of RNA were transcribed using the High Capacity cDNA reverse transcription kit (Applied Biosystems, Darmstadt, Germany). The resulting cDNA was diluted 10-fold and used as template for real-time PCR in an ABI Prism 7300 Sequence detection system (Applied Biosystems, Darmstadt, Germany) using 2.5 µl of the diluted cDNA, TaqMan Universal PCR

Master Mix and TaqMan primer probes (Applied Biosystems, Darmstadt, Germany). The following TaqMan primer probes were used: HNF1A (Hs00167041_m1), HNF4A (Hs00604435_m1), CEBPA (Hs00269972_s1), CEBPB (Hs00270923_s1), CEBPD (Hs00270931), FOXA1 (Hs04187555_m1), FOXA2 (Hs00232764_m1), CAR (Hs00901571_m1), MYB (Hs00920558_m1), E2F5 (Hs00231092_m1), SOX11 (Hs00846583_s1), FOXQ1 (Hs00536425_s1), YBX3 (Hs04195573_u1), all from Applied Biosystems. The PCR conditions were 50°C for 2 min, 95°C for 10 min, followed by 40 cycles of 15 s at 95°C and1 min at 60°C for all PCR reactions. The relative mRNA content was normalized to GAPDH mRNA expression in each sample. For calculations of relative gene expression the $2^{-\Delta\Delta Ct}$ method was used [15]. The expression levels in stem cells and in HLC were normalized to the levels of freshly isolated primary hepatocytes. The results shown correspond to means of three independent cell batches. Results for MRC cells are shown in **Fig. 5**. Results for UKK and CEL cells are shown in **Suppl. Figs. 8 and 9** for liver-enriched and cluster-associated transcription factors respectively.

Immunostaining and fluorescent microscopy

The expression of the hepatocyte differentiation marker albumin was analyzed in stem cells and in HLC by immunofluorescence as previously described [2]. Briefly, media was removed from ESC of HLC (day 17) and the cells were washed twice with PBS. The cells were then fixed in 100% ice cold methanol (-20°C) for 30 min and washed twice with PBS. Prior to staining, the cells were blocked with a solution

of 10% BSA and 0.1% TWEEN in PBS for 1 hour, followed by overnight incubation with mouse anti human albumin antibodies (Sigma, Munchen, Germany) at 4°C. The cells were then washed three times with PBS/Tween 0.1%/BSA 1%, and incubated with alexa fluor 488-labeled secondary antibodies (Life Technologies, Karlsruhe, Germany) for 1h at room temperature. After washing three times in PBS, the fixed cells were incubated with Hoechst 33342 (Molecular Probes, Karlsruhe, Germany) in PBS for 20 min at room temperature. The fixed cells were mounted with PermaFluorTM Aqueous Mounting Medium (Thermo Scientific, Waltham MA, USA) and analyzed in an Olympus TH4-200 microscope, using the Volocity 4 software for acquisition of digital photographs.

Supplemental figures

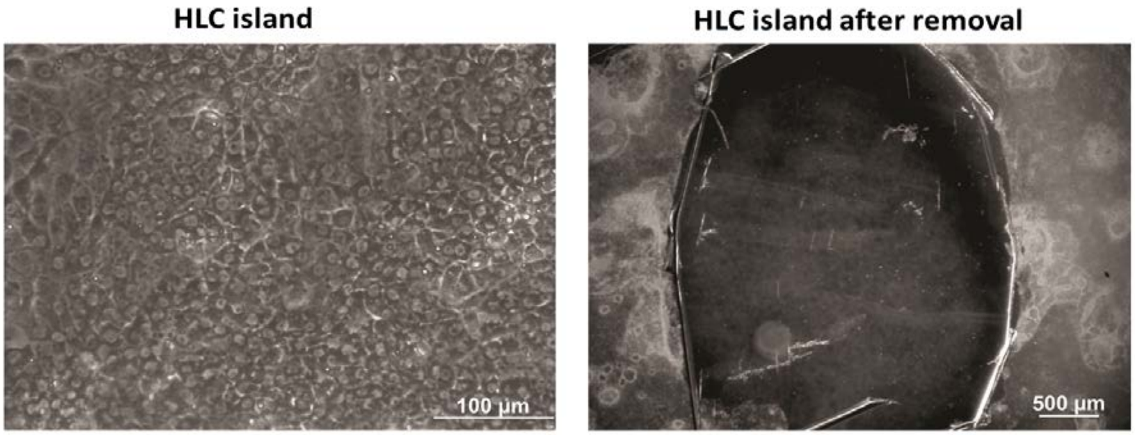

Supplemental Fig. 1: Phase contrast images of hepatic like cell foci (UKK) in culture before and after removal by micropipette.

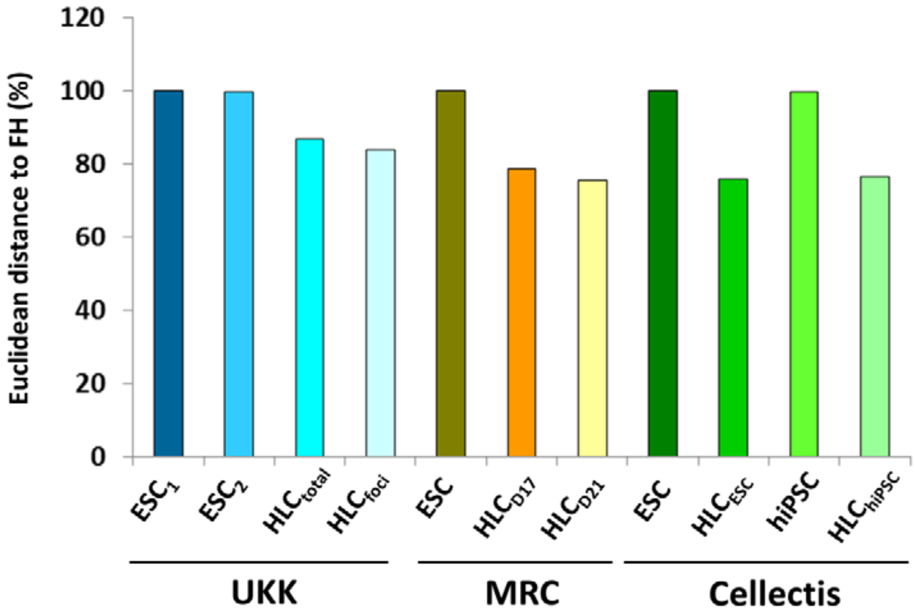

Supplemental Fig. 2: Euclidean distance (ED) between freshly isolated primary human hepatocytes (FH), embryonic stem cells (ESC) and hepatic/like cells (HLC) from UKK, MRC and CEL. The ED was calculated with the statistic toolbox of Matlab (Mathworks Inc., USA), using all differentially expressed genes with a cutoff of 2-fold and a false discovery rate (FDR) adjusted p-value ≤ 0.05. The ED of HLC were scaled to the values of their corresponding ESC, which were set to 100%.

A

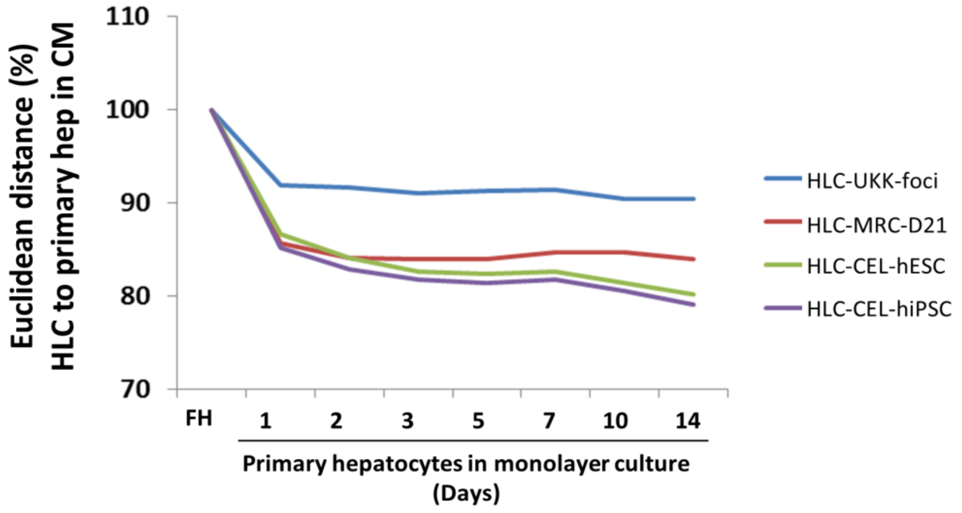

B

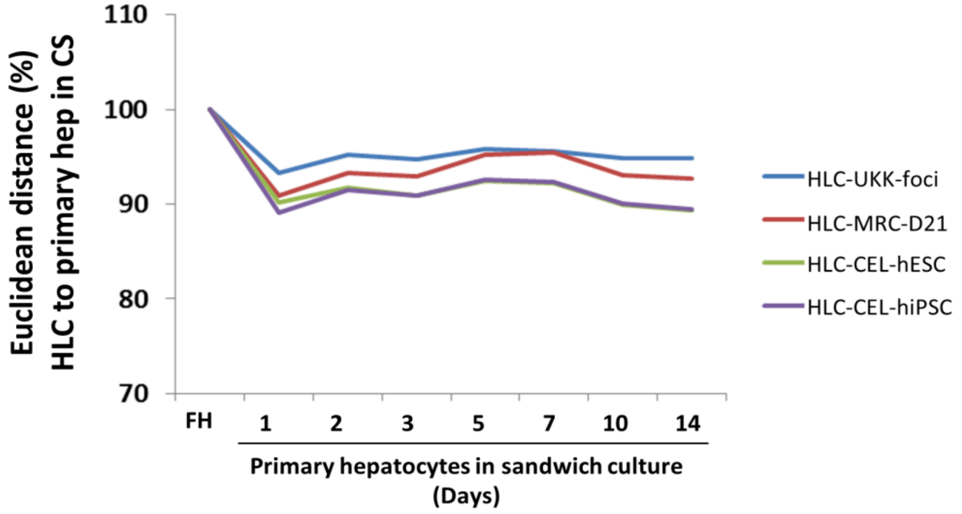

Supplemental Fig. 3: Euclidean distance (ED) between primary hepatocytes (freshly isolated-FH or cultivated in monolayer or sandwich configurations) and HLC from UKK (HLC_{foci}), MRC ($HLC\text{-}MRC_{D21}$), and CEL ($HLC\text{-}CEL_{hESC}$ and $HLC\text{-}CEL_{hiPSC}$). Euclidean distances were calculated using all differentially expressed genes with a cutoff of 2-fold and a (FDR adjusted p-value ≤ 0.05). The ED of cultivated primary hepatocytes were scaled to the values of FH which were defined as 100%.

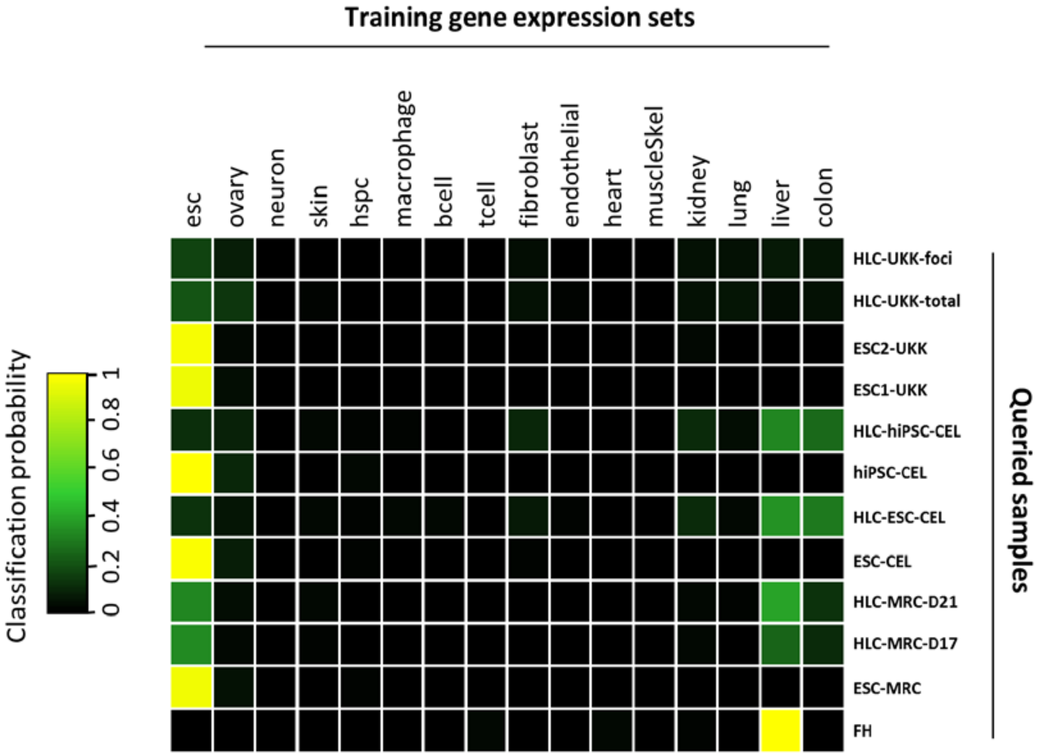

Supplemental Fig. 4: Cell and tissue classification probability of freshly isolated primary hepatocytes (FH), stem cells (ESC) and hepatic-like cells (HLC) from UKK, CEL and MRC. The gene expression profiles of each sample was analyzed with the CellNet algorithm (see supplemental methods) and compared to the training expression profiles defining one of 16 human tissues or cells, as described in Cahan et al [14]. The maximal classification scores were observed for FH and ESC from all centers, corresponding to "liver" and "esc", respectively. HLC from all centers scored three tissues, namely "esc", "liver" and "colon".

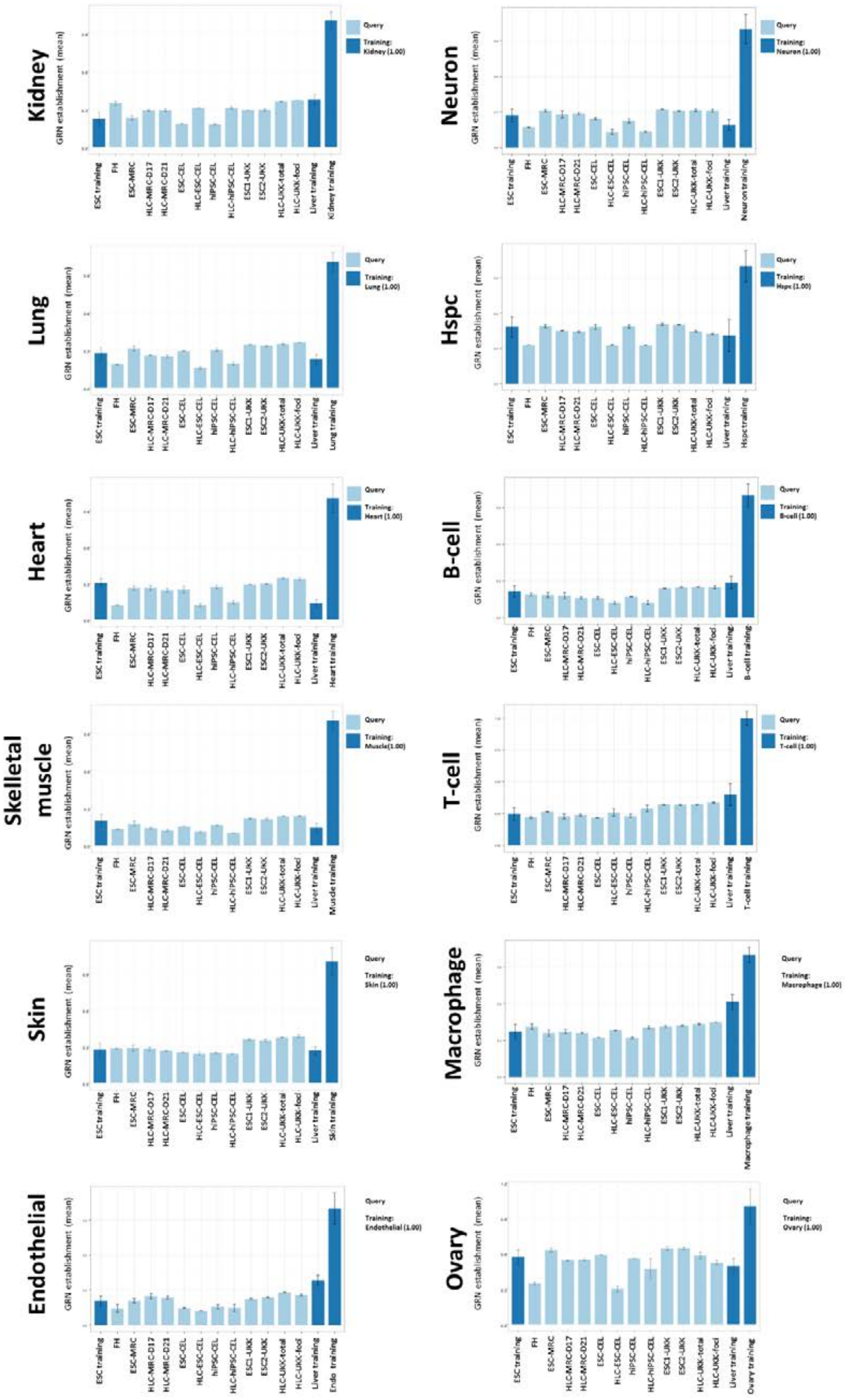
Kidney
Neuron
Lung
Hspc
Heart
B-cell
Skelletal muscle
T-cell
Skin
Macrophage
Endothelial
Ovary
GRN establishment (mean)
Query
Training: Kidney (1.00)
Training: Neuron (1.00)
Training: Lung (1.00)
Training: Hspc (1.00)
Training: Heart (1.00)
Training: B-cell (1.00)
Training: Muscle (1.00)
Training: T-cell (1.00)
Training: Skin (1.00)
Training: Macrophage (1.00)
Training: Endothelial (1.00)
Training: Ovary (1.00)
ESC training
FH
ESC-MRC
HLC-MRC-D17
HLC-MRC-D21
ESC-CEL
HLC-ESC-CEL
hiPSC-CEL
HLC-hiPSC-CEL
ESC1-UKK
ESC2-UKK
HLC-UKK-total
HLC-UKK-foci
Liver training
Kidney training
Neuron training
Lung training
Hspc training
Heart training
B-cell training
Muscle training
T-cell training
Skin training
Macrophage training
Endo training
Ovary training

Supplemental Fig. 5: Establishment of cell or tissue-specific gene regulatory network (GRN) in freshly isolated hepatocytes (FH), stem cells (ESC) and hepatic-like cells (HLC) from UKK, MRC and CEL (in support of Figure 3, main text). Based on the cell/tissue classification analysis, the CellNet algorithm generates a metric for tissue-specific gene regulatory networks, representing a degree of differentiation into one of sixteen human cells or tissues (see Cahan et al [14]). Unlike GRN for “liver”, “colon”, “esc” and “fibroblast” (see Fig. 3), no significant changes were observed for the GRN of the remaining twelve cells or tissues listed here.

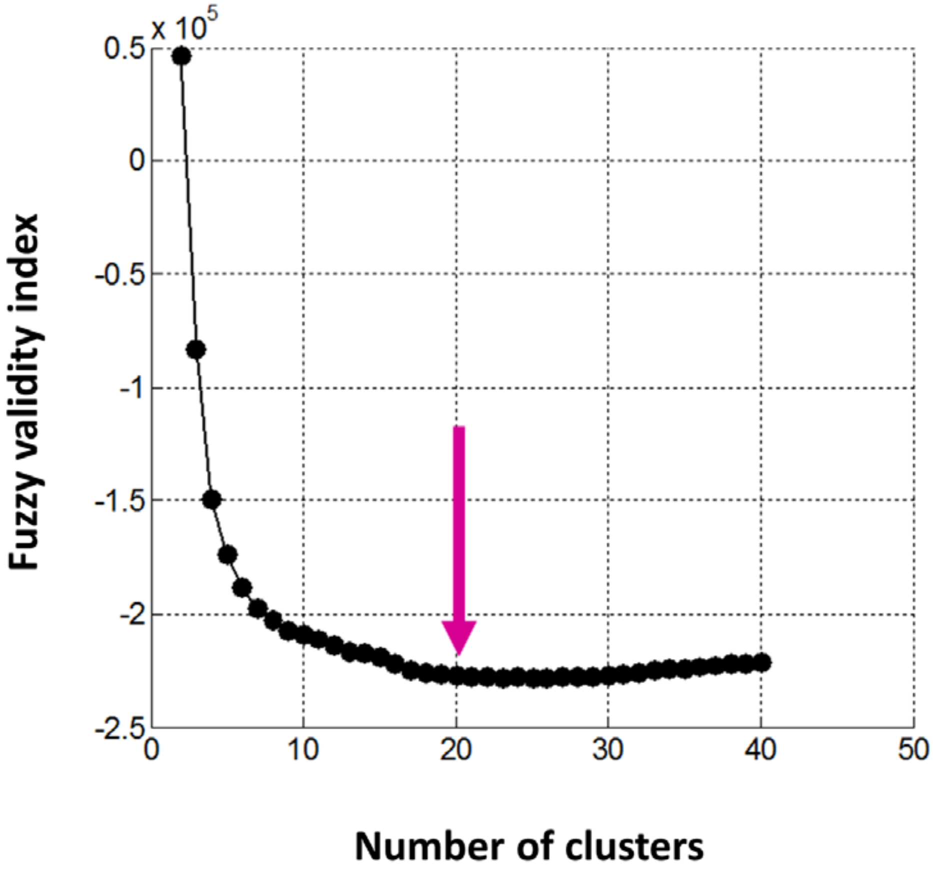

Supplemental Fig. 6: Clustering of gene expression profiles. The fuzzy-c-means algorithm was used to generate groups of genes with similar expression patterns in ESC and HLC [11]. An iterative process establishes a degree of separation (fuzzy validity index) in relation to increasing number of gene clusters. The maximal degree of separation was obtained with twenty clusters (red arrow).

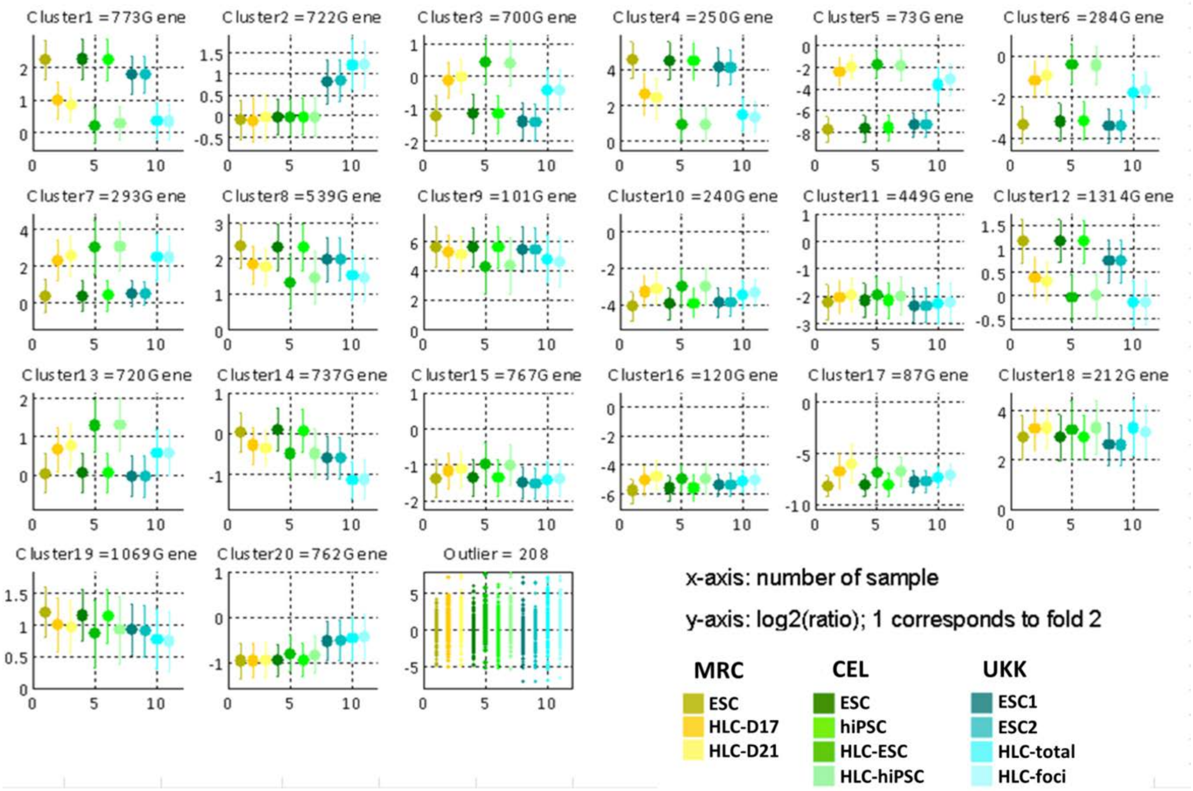

Supplemental Fig. 7: Fuzzy clustering of differentially expressed genes in stem cells and in HLC from three different centers (UKK, MRC and CEL). The time-profiles (i.e. ESC, HLC) of the 10,420 differentially expressed genes were scaled between their respective absolute extreme values. The series were clustered using Fuzzy-c-means [11] with a fuzzy exponent = 1.5, maximum number of iteration 200; minimum cost function improvement = 10-10. The optimal number of clusters was estimated by repeated calculation (number of iterations = 100) of the fuzzy cluster index “separation index” (Suppl. Fig. 6). The analysis generated 20 clusters that contained 10,212 genes, while 208 genes did not fit to any cluster and were classified as outliers.

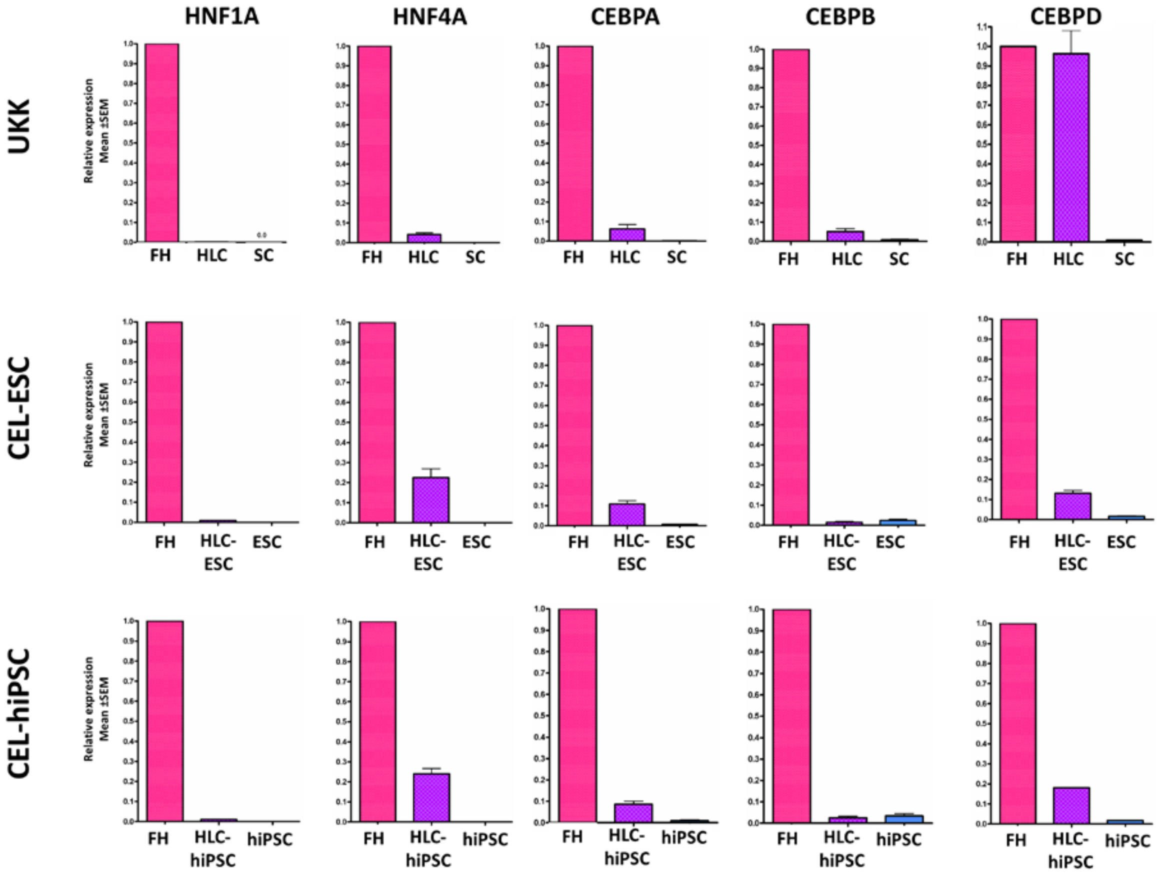

Supplemental Fig. 8: Real time quantitative PCR analysis of liver-enriched transcription factors in ESC, hiPSC and their corresponding HLC from UKK and CEL. The expression levels were normalized to those in freshly isolated primary human hepatocytes (FH). Bars correspond to mean values ± standard error of the mean. Representative of three biological replicas.

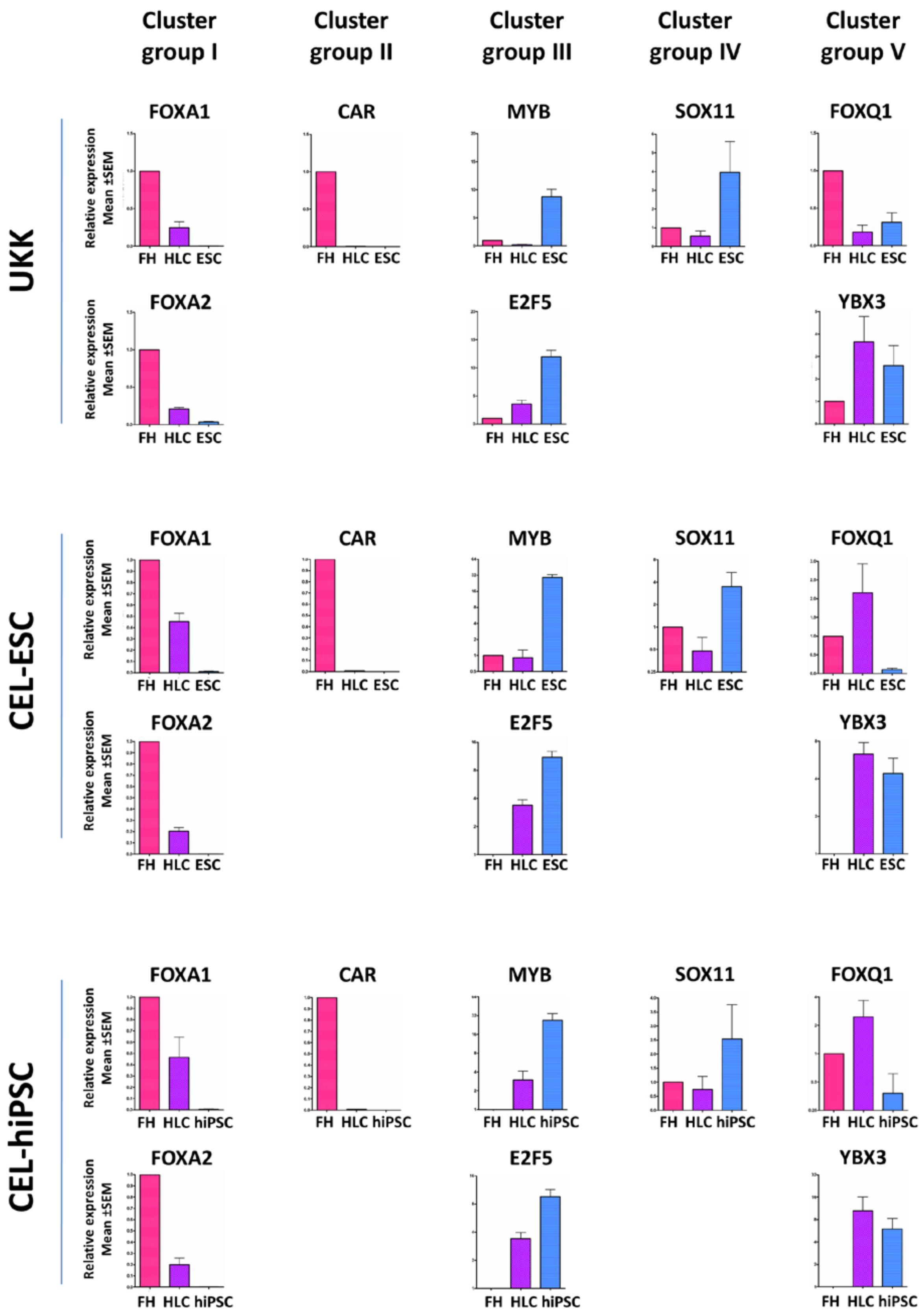
Cluster group I
Cluster group II
Cluster group III
Cluster group IV
Cluster group V
UKK
FOXA1
CAR
MYB
SOX11
FOXQ1
Relative expression Mean ±SEM
FH HLC ESC
FOXA2
E2F5
YBX3
CEL-ESC
CEL-hiPSC
FH HLC hiPSC

Supplemental Fig. 9: Real time quantitative PCR analysis of selected transcription factors representative of cluster groups I to V in ESC, hiPSC and their corresponding HLC from UKK and CEL. The expression levels were normalized to those in freshly isolated primary human hepatocytes (FH). Bars correspond to mean values ± standard error of the mean. Representative of three biological replicas.

Supplemental table legends

Supplemental table 1: Differentially expressed genes in stem cells (ESC) and hepatic-like cells (HLC)

Genes with a fold change ≥ 2-fold in ESC or HLC compared to freshly isolated hepatocytes (FH) (FDR adjusted) are indicated with red (upregulated) or green (downregulated). The fold change and p-value for deregulated genes are indicated for each sample type (i.e. ESC-MRC; HLC-MRC-D17; HLC-MRC-D21). Genes are identified by gene symbol and name.

Supplemental table 2: Differentially expressed genes in primary human hepatocytes cultured in collagen monolayer configuration

Genes with a fold change ≥ 2-fold in cultivated primary hepatocytes compared to freshly isolated hepatocytes (FH) (FDR adjusted) are indicated with red (upregulated) or green (downregulated). The fold change and p-value for deregulated genes are indicated for each time point in culture. Genes are identified by gene symbol and name.

Supplemental table 3: Differentially expressed genes in primary human hepatocytes cultured in collagen sandwich configuration

Genes with a fold change ≥ 2-fold in cultivated primary hepatocytes compared to freshly isolated hepatocytes (FH) (FDR adjusted) are indicated with red

(upregulated) or green (downregulated). The fold change and p-value for deregulated genes are indicated for each time point in culture. Genes are identified by gene symbol and name.

Supplemental table 4: Euclidean distance between FH, ESC and HLC from all centers

The Euclidean distance between FH, ESC and HLC from all centers were calculated as described in Suppl. Methods (page 11), using all differentially expressed genes with a cutoff of 2-fold.

Supplemental table 5: Euclidean distance between FH, cultivated hepatocytes in monolayer or sandwich configurations and HLC

The Euclidean distance between cultivated hepatocytes in monolayer or sandwich configurations, FH and HLC from all centers were calculated as described in Suppl. Methods (page 11), using all differentially expressed genes with a cutoff of 2-fold.

Supplemental table 6: Fuzzy gene clusters

Gene clusters generated as described in Suppl. Methods (page 8). The genes belonging to each cluster are listed in vignettes 1 to 20. Deregulated genes not fitting in any cluster were classified as outliers and listed in vignette “outlier”. Dark

red or green cells indicate fold change ≥ 5. Light red or green cells indicate fold change ≥ 2. Genes are identified by symbol and name.

Supplemental table 7: Gene ontology annotation enrichment in cluster groups

Vignette “GO BKL” contains gene ontology annotations overrepresented in each cluster group. Column A: Gene ontology annotation identification (GO:ID). Column B: GO annotation description. Column C: number of genes (size) belonging to each GO annotation. For each cluster group, the number of genes belonging to the GO annotation list (count) and overrepresentation score (p-value) are indicated (grey cells = p-value<0.05, FDR-adjusted). Vignette “GO BKL (gene)” includes an additional column containing the list of genes identified as belonging to the corresponding GO annotations.

Supplemental table 8: Transcription factors deregulated in cluster groups

Column A: transcription factor identification (TF-ID), according to the TRANSFAC database. Column B: description of the ID annotations. Column C: Number of genes (size) belonging to the corresponding TF-ID. For each cluster group, the number (count) of genes (transcription factors), statistical overrepresentation (p-value) and gene symbols belonging to each TF-IF are indicated.

Supplemental table 9: Transcription factor binding site (TFBS) enrichment in cluster groups

Vignette “GO BKL” contains TFBS overrepresented in each cluster group. Column A: Transcription factor binding site annotation (TRANSFAC database). For each cluster group, the number of genes (count) containing a binding site for the corresponding TF annotation, overrepresentation score (p-value and enrichment) are indicated (grey cells = p-value<0.05, FDR-adjusted). Vignette “GO BKL (gene)” includes an additional column containing the list of genes identified as belonging to the corresponding TF annotations.

Supplemental table 10: Gene ontology annotation enrichment in top 10 to 40% correlated genes between HLC and hepatocytes cultivated in monolayer configuration

Vignette “GO BKL” contains gene ontology annotations overrepresented in each gene group, representing the top 10, 20, 30 and 40% correlated genes between all downregulated genes in primary hepatocytes cultivated in monolayer configuration for 14 days versus all differentially expressed genes in HLC-UKK. Column A: Gene ontology annotation identification (GO:ID). Column B: GO annotation description. Column C: number of genes (size) belonging to each GO annotation. For each cluster group, the number of genes (count) belonging to the GO annotation list and overrepresentation score (p-value) are indicated (grey cells = p-value<0.05, FDR-

adjusted). Vignette "GO BKL (gene)" includes an additional column containing the list of genes identified as belonging to the corresponding GO annotations.

Supplemental table 11: Gene expression values of top 10% correlated genes belonging to "small molecule metabolic process" gene ontology annotation

Expression levels of the 42 genes corresponding to the top 10% correlated genes (table 10) and belonging to the GO:0044281 annotation (small molecule metabolic process). Column A: gene symbol. Column B: gene name. The table shows fold change in cultivated hepatocytes on monolayer (CM) or sandwich (CS) configurations for 14 days, and on HLC from the three research centers.

Ethics committee approval for patient informed consent form for donation of liver resections

EBERHARD KARLS UNIVERSITÄT TÜBINGEN

UKT

Medizinische Fakultät

Ethik-Kommission

Prof. Dr. med. D. Luft
Vorsitzender

Telefon: +49 7071 29-77661
Telefax: +49 7071 29-5965
E-Mail:
ethik.kommission@med.uni-tuebingen.de

Ethik-Kommission an der Medizinischen Fakultät der Eberhard-Karls-Universität und am Universitätsklinikum Tübingen, Gartenstraße 47, 72074 Tübingen

Herrn
Prof. Dr. med. Andreas Nüssler
Berufsgenossenschaftliche Unfallklinik
Schnarrenbergstr. 95
72076 Tübingen

nachrichtlich:
Herrn Prof. Dr. med. Ulrich Stöckle

650/2011BO2 — unsere Projekt-Nummer
23. November 2011 — eingegangen am
13. Dezember 2011 — Datum

Qualitativer und quantitativer Nachweis Xenobiotika - metabolisierender Enzyme unter Berücksichtigung molekularbiologischer Techniken.
Prüfplan („Antrag an das Ethik-Komitee des Klinikums rechts der Isar der Technischen Universität München"), ohne Versionsangabe
Patienteninformation Version 1
Einwilligungserklärung „Wissenschaftliche Verwendung von Blut- und Lebergewebe"
Patienteneinwilligungserklärung (TU München)
Votum der Ethik-Kommission der Fakultät für Medizin der TU München vom 17.10.2007
Begleitschreiben vom 18.11.2011

Sehr geehrter Herr Kollege,

die Unterlagen zur o.g. Studie, für die Sie bereits 2007 eine berufsrechtliche Beratung bei der Ethik-Kommission der Fakultät für Medizin der Technischen Universität München (siehe Schreiben vom 17.10.2007) eingeholt hatten, hat der Ethik-Kommission an der Medizinischen Fakultät und am Universitätsklinikum Tübingen erneut zur Beratung vorgelegen.
Danach bestehen gegen die Weiterführung der obengenannten Studie seitens der Kommission keine Bedenken.
Die Ethik-Kommission empfiehlt Klarstellungen im Prüfplan sowie Änderungen der Patienteninformation und Einwilligungserklärung, die Sie im Folgenden aufgelistet finden.

Prüfplan:

1) Die Angaben zu den Namen, Tätigkeiten und Anschriften der an der Studie beteiligten Ärzte und Wissenschaftler (Abschnitt A 4) sollten überprüft werden.
2) Für den Fall, dass aus Studiengründen serologische Untersuchungen auf HIV, HCV und HBV durchgeführt werden sollten, müsste in der Patienteninformation darauf eingegangen und es müssten die bei einem positiven Befund zu erfüllenden Meldepflichten nach dem Infektionsschutzgesetz beschrieben werden.
3) Der Umgang mit den in der Studie erhobenen Daten muss dargestellt werden (Abschnitt 6) d.h. es müssen Angaben zu Art und Umfang der gespeicherten Daten, die Art der Verschlüsselung, Speicherort, Speicherdauer und Zugangsberechtigte eingefügt werden.

Universitätsklinikum Tübingen
Anstalt des öffentlichen Rechts
Sitz Tübingen
Geissweg 3 · 72076 Tübingen
Telefon +49 7071 29-0
www.medizin.uni-tuebingen.de
Steuer-Nr. 86156/09402
USt-ID: DE 146 889 674

Aufsichtsrat
Hartmut Schrade (Vorsitzender)

Vorstand
Prof. Dr. Michael Bamberg (Vorsitzender)
Gabriele Sonntag (Stellv. Vorsitzende)
Prof. Dr. Karl Ulrich Bartz-Schmidt
Prof. Dr. Ingo B. Autenrieth
Jana Luntz

Banken
Baden-Württembergische Bank Stuttgart
(BLZ 600 501 01) Konto-Nr. 7477 5037 93
IBAN: DE41 6005 0101 7477 5037 93
SWIFT-Nr.: SOLADEST
Kreissparkasse Tübingen
(BLZ 641 500 20) Konto-Nr. 14 144
IBAN: DE79 6415 0020 0000 0141 44
SWIFT-Nr.: SOLADES1TUB

Patientenaufklärung:

1) Die Zahl der Studienteilnehmer sollte genannt werden.
2) Die Angaben zur Blutentnahme sollte überprüft werden. Während im Prüfplan, Abschnitt 4, darauf hingewiesen wird, es würden einmalig 5ml Vollblut während des operativen Eingriffs entnommen, wird in der Patienteninformation beschrieben, diese Entnahme erfolge im Rahmen einer Routineblutuntersuchung (Seite 2) bzw. bei einem ambulanten Termin in der Poliklinik (Seite 3).
3) Eine Speicherung pseudonymisierter Daten bzw. Proben ohne zeitliche Begrenzung ist nicht zulässig ist, d.h. es muss eine maximale Speicherdauer für pseudonymisierte Daten oder Proben festgelegt werden. Danach müssen die Proben/Daten oder die Reidentifikationsliste, die die Zuordnung der Proben/Daten zu den Patienten gestattet, vernichtet werden.
4) Die Kommission weist darauf hin, dass die Verwendung von Patienteninitialen (Patientenaufklärung, TU München) nicht den Anforderungen an eine wirksame Pseudonymisierung entspricht (siehe § 3 Abs 7 LDSG Baden-Württemberg) und deshalb nicht statthaft ist.

Für Rückfragen stehe ich Ihnen gern zur Verfügung.
Mit freundlichen Grüßen

Prof. Dr. med. Dieter Luft
Vorsitzender der Ethik-Kommission

ALLGEMEINE HINWEISE ZUM VOTUM DER ETHIK-KOMMISSION

Mitglieder der Ethik-Kommission

Privatdozent Dr.med. Margitta Albinus	Pharmakologie, Toxikologie
Professor Dr.med. Holger Lerche	Neurologie
Professor Dr.med. Henner Giedke	Psychiatrie
Professor Dr.med. Else Heidemann	Innere Medizin
Professor Dr.med. Jürgen Honegger	Neurochirurgie
Professor Dr.med. Dieter Luft	Innere Medizin
Professor Dr.med. Christian F. Poets	Kinderheilkunde
Professor Dr.iur. Dr.h.c. Georg Sandberger	Medizinorganisationsrecht, Hochschulrecht, deutsches und internationales Wirtschaftsrecht
Professor Dr. Dr. Dr.h.c. Norbert Schwenzer	Zahnheilkunde, Kieferchirurgie
Professor Dr.med. Dr.phil. Urban Wiesing	Medizinische Ethik und Theoretische Medizin

Die Ethik-Kommission an der Medizinischen Fakultät der Universität Tübingen verfährt entsprechend den GCP-/ICH- Richtlinien, der Deklaration von Helsinki in der jeweils gültigen Fassung sowie den gesetzlichen Bestimmungen.

Die Ethik-Kommission ist gemäß § 20 Abs. 7 MPG, Aktenzeichen: Z14-A1871-14924/97, gemäß § 92 Strahlenschutzverordnung, Aktenzeichen: Z 2.1.2-22471/2-EK-012-Ber und gemäß § 28g der Röntgenverordnung, Aktenzeichen: Z 2.1.2-22472/2.EK-013/R registriert.

Die berufsethische und berufsrechtliche Beratung gemäß §15 Abs.1 Berufsordnung für Ärzte in Baden-Württemberg ist für 3 Jahre ab Ausstellungsdatum gültig.

Änderungen im Prüfplan und in der Phase der Umsetzung bitten wir der Kommission mitzuteilen; dabei wären wir Ihnen dankbar, wenn Sie geänderte Passagen deutlich kennzeichnen würden.

Unabhängig vom Beratungsergebnis macht die Ethik-Kommission darauf aufmerksam, dass die medizinische, ethische und rechtliche Verantwortung für die Durchführung einer klinischen Prüfung beim Leiter der klinischen Prüfung und auch bei allen an der Prüfung teilnehmenden Ärzten liegt.

Nach Abschluss der Studie bittet die Kommission um einen abschließenden Bericht.

Supplemental references

[1] Szkolnicka D, Farnworth SL, Lucendo-Villarin B, Hay DC. Deriving functional hepatocytes from pluripotent stem cells. Current protocols in stem cell biology 2014;30:1G 5 1-1G 5 12.

[2] Zhou X, Sun P, Lucendo-Villarin B, Angus AG, Szkolnicka D, Cameron K, et al. Modulating innate immunity improves hepatitis C virus infection and replication in stem cell-derived hepatocytes. Stem cell reports 2014;3:204-214.

[3] Ulvestad M, Nordell P, Asplund A, Rehnstrom M, Jacobsson S, Holmgren G, et al. Drug metabolizing enzyme and transporter protein profiles of hepatocytes derived from human embryonic and induced pluripotent stem cells. Biochemical pharmacology 2013;86:691-702.

[4] Godoy P, Hewitt NJ, Albrecht U, Andersen ME, Ansari N, Bhattacharya S, et al. Recent advances in 2D and 3D in vitro systems using primary hepatocytes, alternative hepatocyte sources and non-parenchymal liver cells and their use in investigating mechanisms of hepatotoxicity, cell signaling and ADME. Archives of toxicology 2013;87:1315-1530.

[5] Godoy P, Hengstler JG, Ilkavets I, Meyer C, Bachmann A, Mueller A, et al. Extracellular Matrix Modulates Sensitivity of Hepatocytes to Fibroblastoid Dedifferentiation and Transforming Growth Factor beta-induced Apoptosis. Hepatology 2009;49:2031-2043.

[6] Zellmer S, Schmidt-Heck W, Godoy P, Weng H, Meyer C, Lehmann T, et al. Transcription factors ETF, E2F, and SP-1 are involved in cytokine-independent proliferation of murine hepatocytes. Hepatology 2010;52:2127-2136.

[7] Bolstad BM, Collin F, Simpson KM, Irizarry RA, Speed TP. Experimental design and low-level analysis of microarray data. Int Rev Neurobiol 2004;60:25-+.

[8] Gentleman RC, Carey VJ, Bates DM, Bolstad B, Dettling M, Dudoit S, et al. Bioconductor: open software development for computational biology and bioinformatics. Genome biology 2004;5:R80.

[9] Smyth GK. Limma: linear models for microarray data. . In: Gentleman R C, V., Dudoit, S., Irizarry, R., Huber, W. , editor. Bioinformatics and Computational Biology Solutions using R and Bioconductor. New York: Springer; 2005. p. 397-420.

[10] Dai M, Wang P, Boyd AD, Kostov G, Athey B, Jones EG, et al. Evolving gene/transcript definitions significantly alter the interpretation of GeneChip data. Nucleic acids research 2005;33:e175.

[11] Bezdek JC, Hathaway RJ. Numerical convergence and interpretation of the fuzzy c-shells clustering algorithm. IEEE transactions on neural networks / a publication of the IEEE Neural Networks Council 1992;3:787-793.

[12] Elkon R, Linhart C, Sharan R, Shamir R, Shiloh Y. Genome-wide in silico identification of transcriptional regulators controlling the cell cycle in human cells. Genome research 2003;13:773-780.

[13] Ulitsky I, Maron-Katz A, Shavit S, Sagir D, Linhart C, Elkon R, et al. Expander: from expression microarrays to networks and functions. Nature protocols 2010;5:303-322.

[14] Cahan P, Li H, Morris SA, Lummertz da Rocha E, Daley GQ, Collins JJ. CellNet: network biology applied to stem cell engineering. Cell 2014;158:903-915.

[15] Livak KJ, Schmittgen TD. Analysis of relative gene expression data using real-time quantitative PCR and the 2(-Delta Delta C(T)) Method. Methods 2001;25:402-408.
